# PFKFB4 facilitates palbociclib resistance in oestrogen receptor‐positive breast cancer by enhancing stemness

**DOI:** 10.1111/cpr.13337

**Published:** 2022-09-20

**Authors:** Sijie Wang, Yuncheng Bei, Qiang Tian, Jian He, Rui Wang, Qiuping Wang, Luchen Sun, Jiangqiong Ke, Congying Xie, Pingping Shen

**Affiliations:** ^1^ Department of Radiation and Medical Oncology The Second Affiliated Hospital and Yuying Children's Hospital of Wenzhou Medical University Wenzhou China; ^2^ State Key Laboratory of Pharmaceutical Biotechnology and The Comprehensive Cancer Center Nanjing Drum Tower Hospital, The Affiliated Hospital of Nanjing University Medical School, School of Life Sciences, Nanjing University Nanjing China; ^3^ Department of Nuclear Medicine Nanjing Drum Tower Hospital, The Affiliated Hospital of Nanjing University Medical School Nanjing China; ^4^ Department of Geriatric Medicine The Second Affiliated Hospital and Yuying Children's Hospital of Wenzhou Medical University Wenzhou China

## Abstract

**Background:**

ER^+^ breast cancer (ER^+^ BC) is the most common subtype of BC. Recently, CDK4/6 inhibitors combined with aromatase inhibitors have been approved by FDA as the first‐line therapy for patients with ER^+^ BC, and showed promising therapeutic efficacy in clinical treatment. However, resistance to CDK4/6 inhibitors is frequently observed. A better understanding of the drug resistance mechanism is beneficial to improving therapeutic strategies by identifying optimal combinational treatments.

**Methods:**

Western blotting, qPCR, flow cytometry and a series of cell experiments were performed to evaluate the phenotype of MCF‐7/R cells. RNA sequencing, non‐targeted metabolomics, shRNA knockdown and tumour cell‐bearing mouse models were used to clarify the drug resistance mechanism.

**Results:**

Here, we found that ER^+^ BC cells have shown an adaptive resistance to palbociclib‐induced cell cycle arrest by activating an alternative signal pathway, independent of the CDK4/6‐RB signal transduction. Continuing treatment of palbociclib evoked cellular senescence of ER^+^ BC cells. Subsequently, the senescence‐like phenotype promoted stemness of ER^+^ BC cells, accompanied by increased chemoresistance and tumour‐initiating potential. Based on transcriptome analysis, we found that PFKFB4 played an important role in stemness transformation and drug resistance. A close correlation was determined between PFKFB4 expression by ER^+^ BC cells and cell senescence and stemness. Mechanistically, metabolomic profiling revealed that PFKFB4 reprogramed glucose metabolism and promoted cell stemness by enhancing glycolysis. Strikingly, diminishing PFKFB4 levels improved drug sensitivity and overcame chemoresistance during palbociclib treatment in ER^+^ BC.

**Conclusions:**

These findings not only demonstrated the novel mechanism underlying which ER^+^ BC cells resisted to palbociclib, but also provided a possible therapeutic strategy in the intervention of ER^+^ BC to overcome drug resistance.

## Graphical abstract

ER^+^ breast cancer (ER^+^ BC) cells have shown an adaptive resistance to palbociclib‐induced cell cycle arrest by activating alternative signal pathway, independent of the CDK4/6‐RB signal transduction. Continuing treatment of palbociclib evoked cellular senescence of ER^+^ BC cells. Subsequently, the senescence‐like phenotype promoted stemness of ER^+^ BC cells, accompanied by increased chemoresistance and tumour‐initiating potential. Based on transcriptome analysis, we found that PFKFB4 played an important role in stemness transformation and drug resistance. A close correlation was determined between PFKFB4 expression by ER^+^ BC cells and cell senescence and stemness. Mechanistically, metabolomic profiling revealed that PFKFB4 reprogramed glucose metabolism and promoted cell stemness by enhancing glycolysis. Strikingly, diminishing PFKFB4 levels improved drug sensitivity and overcame chemoresistance during palbociclib treatment in ER^+^ BC.

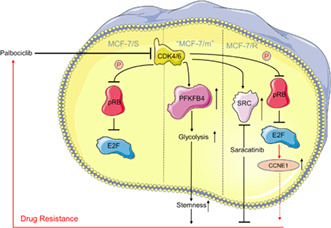

## 1 INTRODUCTION

According to the Global Cancer Observatory, breast cancer (BC) surpassed lung cancer and became the most prevalent type of malignancy worldwide in 2020, accounting for 11.7% of new cancer cases.^1^ To better develop the molecular targeted therapy, BC is classified into several molecular subtypes. Among them, luminal A (hormone receptor [HR]^+^/human epidermal growth factor receptor 2 [HER2]^−^) is the most common subtype.^2^ Currently, the cyclin‐dependent kinase (CDK) 4/6 inhibitor combined with an aromatase inhibitor is the first‐line therapy for patients with HR^+^, HER2^−^ advanced BC.^3^, ^4^

CDK4 and CDK6 play a pivotal role in regulating cell proliferation.^5^ They can interact with D‐type cyclins and hyperphosphorylate retinoblastoma‐associated protein 1 (RB1), which in turn promotes the cell‐cycle transition from G1 to S phase in cancer cells. Recently, it has been reported that CDK4/6 pathway was highly activated in ER^+^ BC cells, which promoted cell proliferation, migration and angiogenesis.^6^, ^7^, ^8^ The increased levels of cyclin D1 and CDK4/6 activity are the major reason for oestrogen to be mitogenic and drive cell proliferation in ER^+^ BC cells. Given the finding that inhibiting CDK4 and CDK6 might retard cancer cell development, several CDK4/6 dual‐inhibitors come into being and show both good preclinical and clinical outcomes.^9^ Fortunately, several ATP‐competitive inhibitors of CDK4/6 have been approved and utilized in clinical applications, including palbociclib, ribociclib and abemaciclib.^10^, ^11^, ^12^

Clinically, palbociclib plus letrozole can significantly prolong progression‐free survival (NCT01740427).^13^, ^14^ Moreover, CDK4/6 inhibitors combined with fulvestrant are universally recommended as second‐line therapy.^15^, ^16^ Palbociclib (PD0332991) is an oral and highly selective inhibitor of CDK4/6, which leads to cell‐cycle arrest by inhibiting the phosphorylation of RB1. Several preclinical and clinical studies have shown that palbociclib effectively delayed the proliferation of ER^+^ BC cells.^17^ However, clinical resistance to CDK4/6 inhibitors is now a general problem and needs to be solved urgently.^18^ For instance, in the reported PALOMA‐2 trial, nearly 1/3 of patients experienced recurrence on CDK4/6 inhibitors within 2 years. Therefore, a better understanding of the drug resistance mechanism is beneficial to improving therapeutic strategies by identifying optimal combinational treatments.

The molecular mechanism of resistance to CDK4/6 inhibitors is summarized and classified as follows: (1) pathway reactivation, including increased activity of the drug target and hyper‐activation of downstream effectors such as downstream kinase CDK2; (2) pathway bypass, for example, activation of mTOR signalling as an alternate pathway.^19^ The current understanding of the mechanisms of CDK4/6 inhibitor resistance is still far from complete. As research has progressed, some researchers have proposed the term ‘pathway indifference’.^20^ It means an alternative cell state that is independent of the index oncogenic pathway. Despite continued suppression of the index drug target and its downstream effectors, the specific phenotype confers drug resistance.

6‐Phosphofructo‐2‐kinase/fructose‐2, 6‐biphosphatase 4 (PFKFB4) is a bi‐functional enzyme, which possesses both kinase and phosphatase activity. PFKFB4 dynamically modulates the synthesis and hydrolysis of fructose‐2,6‐biphosphate (F2,6BP), which is an allosteric glycolytic regulator.^21^ F2,6BP allosterically activates phosphofructokinase1 (PFK1), a critical rate‐limiting enzyme in glycolysis, thereby enabling glycolytic flux to lactate. PFKFB4 was first identified in the testes and has been found to be widely expressed in a variety of organs. Recently, it has been reported that PFKFB4 is associated with cancer proliferation, metastasis and progression in multiple tumour cell types.^22^, ^23^, ^24^ Although PFKFB4 has been shown to be an anti‐tumour target, its relationship with chemoresistance is still not clear.

Of note, increasing studies have reported that continuous prolonged exposure to CDK4/6 inhibitors, such as palbociclib, ultimately induces resistant cell populations. A few mechanisms have been reported to be associated with this long‐term acquisition of CDK4/6 inhibitor resistance, including the alterations in CDK4/6‐Rb and CDK2 signalling pathways. Recently, CDK4/6 inhibition is reported to cause a profound G1 cell cycle arrest in Rb^+^ cells, but this phenomenon is transiently in some cancer models.^6^ Interestingly, this ‘adaptive response’ has been shown to be a major cause of drug resistance or the durability of therapeutic response. In ER^+^ BC cells, Herrera‐Abreu et al. found that palbociclib treatment caused a temporary cell cycle arrest and documented a novel therapy, which combined palbociclib and PI3K inhibitor, to inhibit this adaptive response. Thus, more studies, developing the integration of CDK4/6 inhibition into the adjuvant combinatorial settings, need to be carried out to better block early adaptive response and induce more potent cell cycle arrest. Here, we sought to interrogate the underlying mechanism of CDK4/6 inhibitor resistance and seek a promising drug combination with CDK4/6 inhibitors to overcome this resistance.

## 2 MATERIALS AND METHODS

### 2.1 Reagents

Palbociclib, saracatinib, KAN0438757 and stattic were purchased from MCE. Iodoacetamide was purchased from Sigma‐Aldrich.

### 2.2 Cell lines and cell culture

Human embryonic kidney 293T and human BC MCF‐7 cell lines were purchased from the Cell Bank of the Chinese Academy of Sciences in Shanghai. All cell lines were maintained in Dulbecco's modified Eagle's medium (DMEM) supplemented with 1% penicillin–streptomycin and 10% fetal bovine serum (FBS; Gibco, mycoplasma contamination check was carried out by our group). All cells were cultured at 37°C with 5% CO_2_.

### 2.3 Mouse studies

Cell lines were inoculated subcutaneously into 6‐ to 8‐week‐old female nude mice (Model Animal Research Center of the Nanjing University) and bred in our animal facilities under specific pathogen‐free conditions. Animal care and experiments for this study were approved by Institutional Animal Care and Use Committee, Nanjing University (Accreditation no. IACUC‐2109010). For xenograft cell transplant models, the nude mice were inoculated hypodermically with 2 × 10^7^ cells suspended in 100 μl phosphate‐buffered saline (PBS). According to animal care and enforcement, the largest subcutaneous tumour mass on one flank was <1 cm^3^. Tumour volumes were monitored daily with the vernier calliper, and the volume (0.5 ×  long diameter × short diameter × short diameter) was calculated. Treatment was initiated when tumours became palpable on Day 7 ending on Day 17 post tumour implant. The CDK4/6 inhibitor palbociclib was administered by intragastric injection daily at 100 mg/kg.^25^

### 2.4 Immunohistochemistry, RT‐qPCR and western blot

For details about immunofluorescence, RT‐qPCR and western blot were performed according to our previous publication.^26^ The primers used are listed in Table 1. Antibodies used in these experiments are listed in Table 2.

**TABLE 1:** q‐PCR primer

beta‐Actin
Forward GGCTGTATTCCCCTCCATCG
Reverse CCAGTTGGTAACAATGCCATGT
GAPDH
GGAGCGAGATCCCTCCAAAAT
GGCTGTTGTCATACTTCTCATGG
ALDH1
Forward ATACTTGTCGGATTTAGGAGGCT
Reverse GGGCCTATCTTCCAAATGAACA
FGFR1
Forward TAATACCACCGACAAGGAAATGG
Reverse TGATGGGAGAGTCCGATAGAGT
NOTCH1
Forward GATGGCCTCAATGGGTACAAG
Reverse TCGTTGTTGTTGATGTCACAGT
OCT4
Forward GGCTTCAGACTTCGCCTCC
Reverse AACCTGAGGTCCACAGTATGC
SOX1
Forward AAGGAACACCCGGATTACAAGT
Reverse GTTAGCCCAGCCGTTGACAT
MMP‐2
Forward CAAGTTCCCCGGCGATGTC
Reverse TTCTGGTCAAGGTCACCTGTC
MMP‐9
Forward CTGGACAGCCAGACACTAAAG
Reverse CTCGCGGCAAGTCTTCAGAG
E‐cadherin
Forward CAGGTCTCCTCATGGCTTTGC
Reverse CTTCCGAAAAGAAGGCTGTCC
N‐cadherin
Forward AGCGCAGTCTTACCGAAGG
Reverse TCGCTGCTTTCATACTGAACTTT
Vimentin
Forward CGTCCACACGCACCTACAG
Reverse GGGGGATGAGGAATAGAGGCT
Slug
Forward TGGTCAAGAAACATTTCAACGCC
Reverse GGTGAGGATCTCTGGTTTTGGTA
Snail
Forward CACACGCTGCCTTGTGTCT
Reverse GGTCAGCAAAAGCACGGTT
Twist1
Forward GGACAAGCTGAGCAAGATTCA
Reverse CGGAGAAGGCGTAGCTGAG
CCND1
Forward GCTGCGAAGTGGAAACCATC
Reverse CCTCCTTCTGCACACATTTGAA
RB1
Forward CTCTCGTCAGGCTTGAGTTTG
Reverse GACATCTCATCTAGGTCAACTGC
E2F1
Forward ACGCTATGAGACCTCACTGAA
Reverse TCCTGGGTCAACCCCTCAAG
CDK2
Forward CCAGGAGTTACTTCTATGCCTGA
Reverse TTCATCCAGGGGAGGTACAAC
CCND3
Forward TACCCGCCATCCATGATCG
Reverse AGGCAGTCCACTTCAGTGC
JUN
Forward TCCAAGTGCCGAAAAAGGAAG
Reverse CGAGTTCTGAGCTTTCAAGGT
MYB
Forward GAAAGCGTCACTTGGGGAAAA
Reverse TGTTCGATTCGGGAGATAATTGG
MYBL2
Forward CCGGAGCAGAGGGATAGCA
Reverse CAGTGCGGTTAGGGAAGTGG
CCNA1
Forward GAGGTCCCGATGCTTGTCAG
Reverse GTTAGCAGCCCTAGCACTGTC
CCNE1
Forward AAGGAGCGGGACACCATGA
Reverse ACGGTCACGTTTGCCTTCC
CCNE2
Forward TCAAGACGAAGTAGCCGTTTAC
Reverse TGACATCCTGGGTAGTTTTCCTC
P12
Forward ATGTCTTACAAACCGAACTTGGC
Reverse GCCCGTAGTCACTGAGCAG
P16
Forward GATCCAGGTGGGTAGAAGGTC
Reverse CCCCTGCAAACTTCGTCCT
P21
Forward TGTCCGTCAGAACCCATGC
Reverse AAAGTCGAAGTTCCATCGCTC
AREG
Forward GTGGTGCTGTCGCTCTTGATA
Reverse CCCCAGAAAATGGTTCACGCT
BMP7
Forward TCGGCACCCATGTTCATGC
Reverse GAGGAAATGGCTATCTTGCAGG
PPARD
Forward CAGGGCTGACTGCAAACGA
Reverse CTGCCACAATGTCTCGATGTC
PPARG
Forward GGGATCAGCTCCGTGGATCT
Reverse TGCACTTTGGTACTCTTGAAGTT
PFKFB4
Forward TCCCCACGGGAATTGACAC
Reverse GGGCACACCAATCCAGTTCA
PRAME
Forward AGCCTTTGACGGGAGACAC
Reverse GAGTTCTTCCGTAAATCCAGCA
TEX19
Forward TCTACGCCTCCTGGATGTATC
Reverse CAGACCTGCATCTTCCAACCC
TIMP1
Forward CTTCTGCAATTCCGACCTCGT
Reverse ACGCTGGTATAAGGTGGTCTG
TRIP6
Forward CCTGGACGCCGAGATAGACT
Reverse CGGTAGTGTAAGAGGCTGGA

**TABLE 2:** Antibody

Anti‐ALDH1	Bioss	bs‐10162R
Anti‐E‐cadherin	Santa Cruz Biotechnology	sc‐7870
Anti‐Vimentin	Bioss	bsm‐33170M
Anti‐b‐Actin‐HRP conjugated	Abclonal	AC028
Anti‐GAPDH‐HRP conjugated	KANG CHEN	KC‐5G5
OCT4	Bioss	bs‐0830R
CDK4	Santa Cruz Biotechnology	sc‐56277
Phospho‐CDK4‐T172	Abclonal	AP0593
CCND1	Bioss	bs‐0623R
RB1	Bioss	bs‐2777R
Phospho‐ RB‐S780	Bioss	bs‐1347R
PFKFB4	Zhengneng Biotechnology	861412
CD44v	Our laboratory	[26]
Anti‐PD‐L1 PE‐conjugated	BD Biosciences	558091
Anti‐CD44 APC‐Cy7‐conjugated	BioLegend	103027
Anti‐CD24 PE‐conjugated	BD Biosciences	553262

### 2.5 Flow cytometry

All cells were pelleted and washed with FACS buffer (PBS supplemented with 2% FBS and 1 mM EDTA), and pre‐incubated with Fc receptor blocker (FcR Blocking Reagent, Miltenyi Biotec) for 15 min at 4°C. Cells were then incubated with Fluorescent antibodies for a further 30 min at 4°C and were washed two times with ice‐cold FACS buffer. All flow cytometry experiments were performed with the BD FACS calibre instrument. Data were analysed with the NovoExpress software.

### 2.6 Mammosphere formation assay

Six‐well plates were pre‐coated with 1 ml poly (2‐hydroxyethyl methacrylate, pHEMA) (12 g pHEMA was dissolved in 1 L 95% ethanol) at 37°C for 48–72 h. MCF‐7 cells were pelleted and resuspended in DMEM/F12 media (Gibco). The number of living cells was calculated, and then the cells were plated at a density of 1 × 10^4^ cells per well in 2 ml DMEM media containing insulin, transferrin, selenium (ITS) and 20 ng ml^−1^ EGF (Sigma‐Aldrich). After 10–14 days, culture of MCF‐7 cells at 37°C with 5% CO_2_, and Spheres greater than 50 μm were subsequently counted.

### 2.7 Pro‐invasion assay

MCF‐7 cells were seeded in Matrigel‐coated invasion chambers (12 wells, 16 mm pore size) and supplemented with DMSO, Palbociclib. After 12–24 h, cells were fixed and stained with crystal violet, and then invading cells were counted.

### 2.8 Analysis of the cellular senescence

The senescence of MCF‐7 cells was evaluated by detecting the activity of β‐galactosidase (SA‐β‐gal) with Senescence β‐Galactosidase Staining Kit (Beyotime), and the experience was according to the manufacturer's instruction. Detail briefly, MCF‐7 cells were washed with PBS, fixed with fixing solution at room temperature for 15 min, washed three times with PBS, and then maintained with the staining solution at 37°Cfor 8–10 h, and then senescent cells were counted.

### 2.9 Cell viability assay

All cells were seeded in the 96‐well plates (1 × 10^3^ cells/well). The cell viability was detected by MTT assay according to the manufacturer's instruction, and then was expressed as relative cell viability with the OD_570_ values. The experiment was done in three repetitions for each group.

### 2.10 Cell apoptosis analysis

Cell apoptosis test was performed using Annexin V‐FITC/PI double staining Apoptosis Detection Kit (Vazyme) and was according to the manufacturer's instruction by flow cytometry. Detail briefly, cells were trypsinized and washed with PBS, then centrifuged and resuspended with 400 μl of Annexin binding buffer. Cells were maintained with 5 μl of FITC staining solution and 5 μl of PI staining solution at room temperature for a total of 15 min. The samples were examined within 1 h by flow cytometry.

### 2.11 Quantification of metabolites by LC–MS/MS

To quantify polar metabolite concentrations, we used the Agilent 1200 Series HPLC instrument (Agilent) combined with an autosampler, quaternary pump and vacuum degasser. Chromatographic peaks were separated on an ACE Excel 3 C18 column (100 mm × 2.1 mm, 3.0 μm) at the temperature of 40°C. The mobile phase composed of 0.1% formic acid aqueous solution (A) and acetonitrile containing 0.1% formic acid (B) was adopted as follows: 0–2 min, 5% B; 2–7 min, 5%–65% B; 7–20 min, 65%–95% B; 20–22 min, 95% B; 22–23 min, 95%–5% B; 23–28 min, 5% B. About 10 μl of each plasma sample was pooled into the quality control (QC) sample to assess the stability of the developed method during the whole analysis. Identification of potential biomarkers and analysis of related metabolic pathways were processed by XCMS Online (https://xcmsonline.scripps.edu/), Human Metabolome Database (HMDB, http://www.hmdb.ca/spectra/ms/search), KEGG (http://www.genome.jp/kegg/), SMPDB (https://smpdb.ca/) and MetaboAnalyst (http://www.metaboanalyst.ca/). The related analysis was performed as previously described.^27^

### 2.12 Pan‐cancer gene expression analysis

We used the ‘Expression analysis‐Profile’ module of the GEPIA2 (Gene expression profiling interactive analysis, version 2) web server (http://gepia2.cancer-pku.cn/#analysis) to obtain the plots of the expression difference between the multiple tumour tissues and the corresponding normal tissues of the GTEx (Genotype‐tissue expression) database, under the settings of *q*‐value cutoff = 0.01, |log2FC| (Fold change) cutoff = 1, and ‘Match TCGA normal and GTEx data’.

### 2.13 RNA sequence and data analysis

RNA sequencing between the control MCF‐7 and MCF‐7m cells was performed by BGISEQ platform. The related analyses, including Venn, heatmap and GO enrichment analysis, were accomplished by BGI Dr. Tom system and BioJupies online tool. Gene Set Enrichment Analysis was performed as previously described.^26^

### 2.14 Statistical analysis

All statistical analyses were generated based on at least three independent experiments and performed by GraphPad Prism 8.0 (GraphPad). Differences groups included two‐tailed unpaired Student's *t*‐test, and one‐ or two‐way analysis of variance (ANOVA). Differences were set as significant at *p* < 0.05. Unless indicated in the figure legend, all data are shown as mean ± SEM.

## 3 RESULTS

### 3.1 Palbociclib‐resistant MCF‐7 cell exhibits a more aggressive phenotype compared to normal MCF‐7 cell

Palbociclib‐resistant MCF‐7 cells (hereafter referred to as MCF‐7/R) were generated by exposing palbociclib‐sensitive parental cells (henceforward, MCF‐7/S) to constant concentration (1 μM) of palbociclib until drug resistance was achieved (Figure S1A).^28^ Comparing palbociclib‐resistant MCF‐7 cells with parental MCF‐7 cells, there was no significant difference in cell morphology (Figure S1B). Then, the cytotoxic effect of palbociclib on MCF‐7/S and MCF‐7/R cells was assessed by 3‐(4,5‐dimethylthiazol‐2‐yl)‐2,5‐diphenyltetrazolium bromide (MTT) assay. Both groups were exposed to different concentrations of palbociclib for 120 h or maintained under drug‐free conditions and the cytotoxic effect of the drug was evaluated (Figure S1C). Cell viability decreased dose‐dependently in both the treatment group and the control group. However, when the concentrations ranged from 10 nM to 1 μM, the percentage viability of MCF‐7/R decreased at lower rates compared with controls. Palbociclib reduced the percentage viability of MCF‐7/R cells to ~80% at a concentration of 1 μM, whereas MCF‐7/S cells were only <40%. This result indicates that the subline MCF‐7/R has stable resistance to different concentrations of palbociclib.

To further evaluate the palbociclib resistance of our cell model, we analysed the effect of palbociclib on the cell cycle of MCF‐7/S and MCF‐7/R cells by propidium iodide (PI) staining. As shown in Figure S1D, PI staining of MCF‐7/S cells after cultivation with different concentrations of palbociclib for 24 h increased the number of cells in G1 phase compared with no inhibitor. The higher the concentration of palbociclib was, the larger the number of MCF‐7/S cells arrested in G1/S increased significantly. However, there was no significant increase in the number of MCF‐7/R cells in G1 phase after culture with higher doses of palbociclib. For example, when palbociclib ranged from 10 to 100 nM, MCF‐7/S cells arrested in G1 phase of the cell cycle had a 20% increase, whereas MCF‐7/R increased only by 3%. We next investigated whether MCF‐7/R cells were stably palbociclib‐resistant if we prolonged the time of drug treatment. MCF‐7/S and MCF‐7/R cells were cultured with 100 nM palbociclib for different times (24, 48 and 72 h). PI staining showed that the number of MCF‐7/S cells arrested in G1 phase after treatment with palbociclib increased in a time‐dependent manner, while MCF‐7/R cells achieved palbociclib‐resistance regardless of medication time (Figure S1E,F). To sum up, MCF‐7/R could model palbociclib resistance and we used the drug‐resistant model for further study.

**FIGURE 1:**
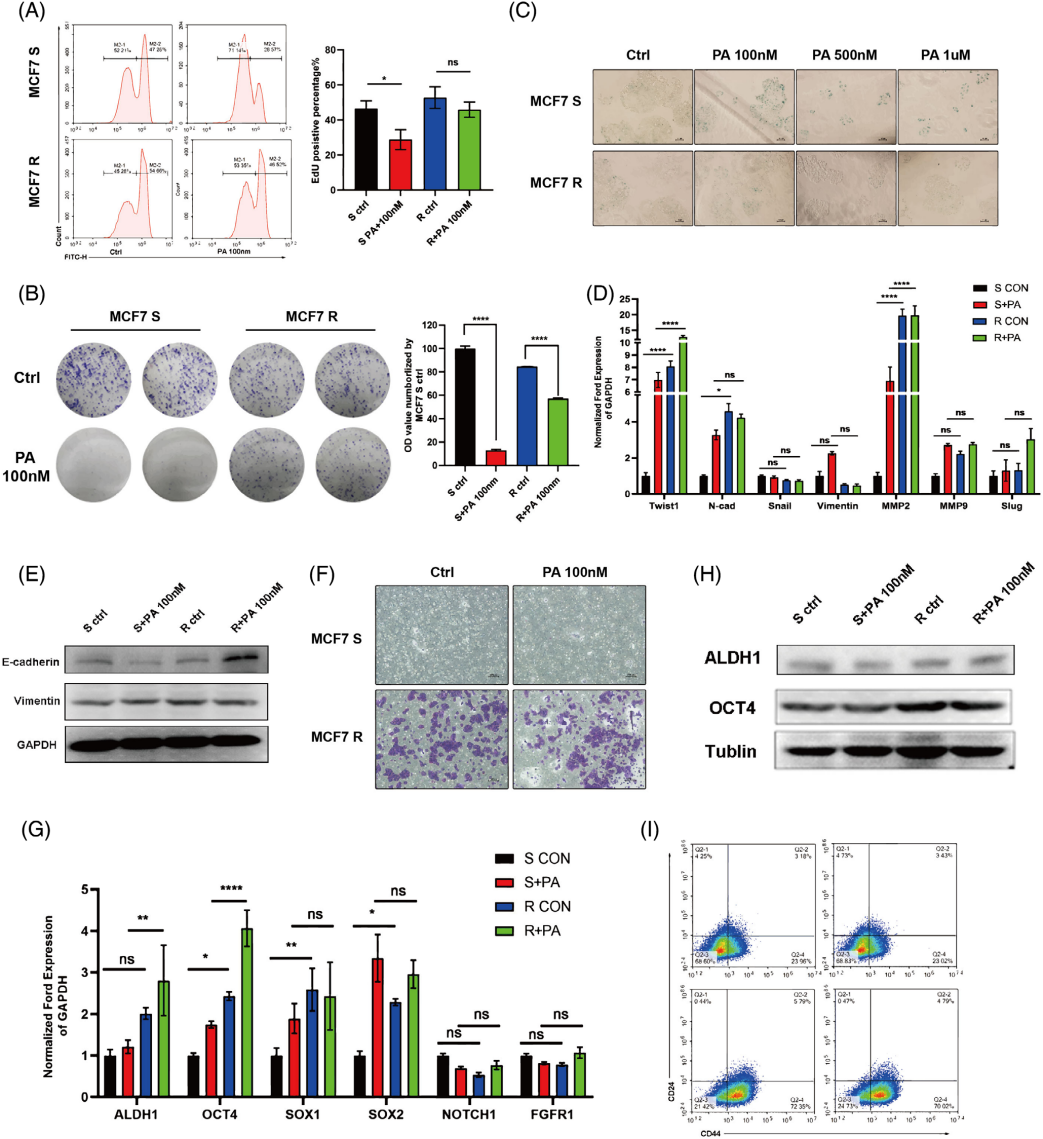
MCF‐7/R cell exhibits a more aggressive phenotype compared to normal MCF‐7/S cell. (A) The cell proliferation of MCF‐7/S and MCF‐7/R cells was detected by the EdU proliferation assay. (B) Colony formation assay. MCF‐7/R cells showed stronger proliferative ability than MCF‐7/S cells, after treatment with 100 nM palbociclib. Right, quantitative analysis of colonies. (C) SA‐β‐Gal staining of MCF‐7/S and MCF‐7/R cells 7 days after treatment of different concentrations of palbociclib. Scale bars, 100 μm. (D) RT‐qPCR detection of the expression of EMT‐related signature genes (Twist1, E‐cadherin, N‐cadherin, Snail, Vimentin, MMP9 and Slug) at the mRNA level in MCF‐7/S and MCF‐7/R cells with or without palbociclib treatment. The results of statistical significance analysis (MCF‐7/S vs. MCF‐7/R, MCF‐7/S + PA vs. MCF‐7/R + PA) were marked on the figure. (E) Immunoblot analysis of invasion marker, E‐cadherin and Vimentin, expression in MCF‐7/S and MCF‐7/R cells. Tubulin and GAPDH were analysed as loading controls, respectively. (F) MCF‐7/S and MCF‐7/R cells were treated with 100 nM palbociclib, and followed by examining the invasion ability via Transwell invasion assay. (G) mRNA level of CSC markers (ALDH1, OCT4, SOX1, SOX2, NOTCH1 and FGFR1) expression in MCF‐7/S and MCF‐7/R cells. The results of statistical significance analysis (MCF‐7/S vs. MCF‐7/R, MCF‐7/S + PA vs. MCF‐7/R + PA) were marked on the figure. (H) Immunoblot analysis of ALDH1 and OCT4 expression in cells depicted in (G). (I) Representative flow cytometric analysis of CD44+CD24−/low BCSC population in MCF‐7/S and MCF‐7/R cells. Data represent mean ± SEM. The experiments were repeated at least two times to observe concordant statistical significance. ns, no significance, *p < 0.05, **p < 0.01, ***p < 0.001, ****p < 0.0001.

After the palbociclib‐resistant MCF‐7 cell line model was established, we evaluated the effect of palbociclib on cell proliferation and apoptosis and the differences in cell invasion, stemness and senescence of MCF‐7/S and MCF‐7/R cells. The 5‐ethynyl‐2′‐deoxyuridine (EdU) assay validated that the proliferation capacity of MCF‐7/S cells was decreased significantly in response to 100 nM palbociclib while MCF‐7/R cell proliferation was hardly affected (Figure 1A). A colony formation assay was performed to detect the effect of palbociclib on the clonogenic capacity of MCF‐7/S and MCF‐7/R cells. The results showed that the colony formation rate of MCF‐7/S cells treated with 100 nM palbociclib was significantly lower than that of the untreated group (Figure 1B). Although it was reported that palbociclib was not able to induce apoptosis, we analysed cell apoptosis by Annexin V‐FITC and PI staining to evaluate the cell state of MCF‐7/R cells. As expected in Figure S2A,B, palbociclib did not induce apoptosis of MCF‐7/S and MCF‐7/R cells regardless of the drug concentration. In addition, we observed that palbociclib only induced cell senescence in MCF‐7/S cells and failed to trigger senescence in MCF‐7/R cells, which indicated that MCF‐7/R cells were able to resist CDK4/6 inhibitor‐induced senescence (Figure 1C).

Epithelial–mesenchymal transition (EMT) is a major factor in promoting cancer progression. Several studies have shown that EMT is linked with metastasis and contributes to tumour heterogeneity and therapeutic resistance.^29^, ^30^ Furthermore, invasiveness and EMT were detected in both the MCF‐7/S and MCF‐7/R groups using q‐PCR, western blot and Transwell analyses. The results indicated that twist1, N‐cadherin and MMP2 mRNA expression levels in MCF‐7/R cells were significantly higher than those in MCF‐7/S cells (Figure 1D). In the MCF‐7/R group, vimentin protein levels were upregulated, while E‐cadherin protein levels were downregulated (Figure 1E). The Transwell results showed that the invasion capability of MCF‐7/R cells was stronger than that of MCF‐7/S cells, which was consistent with the above‐mentioned results (Figure 1F). Additionally, we considered that MCF‐7/R cells exhibited EMT state according to the detection of EMT‐related markers.

EMT plays an essential role in generating invasive mesenchymal cells and CSCs. The discovery of the connection between EMT and stemness has reported that BC cells seem to turn into a stem cell‐like state after they undergo an EMT.^31^ To detect the stemness of MCF‐7/S and MCF‐7/R cells, we used real‐time PCR, western blot and immunofluorescence analyses. Both the mRNA expression levels and protein levels of ALDH1 and OCT4 were dramatically increased in the MCF‐7/R group, compared with the MCF‐7/S group (Figure 1G,H). The flow cytometric results indicated that CD24 negative and CD44 positive subpopulation accounted for a larger proportion in the MCF‐7/R group (Figure 1I).

Recently, studies have indicated that CDK4/6 inhibitors may regulate the expression of PD‐L1 in tumour cells. It provides a theoretical basis for proposing the combination therapy with CDK4/6 and anti‐PD‐L1 inhibitors. As mentioned above, we examined the expression of PD‐L1 in MCF‐7/S and MCF‐7/R cells after treatment with palbociclib. However, there was no aberrant expression of PD‐L1 in both MCF‐7/S and MCF‐7/R cells (Figure S2C).

In summary, MCF‐7/R has a more aggressive phenotype, including stronger proliferative capacity, higher migratory and invasion ability. Additionally, MCF‐7/R tends to induce a more mesenchymal‐like phenotype and acquires novel stem‐cell features.

### 3.2 ER ^+^ BC cells showed an adaptive resistance to palbociclib‐induced cell cycle arrest by activating an alternative signal pathway, independent of the CDK4/6‐RB signal transduction

**FIGURE 2:**
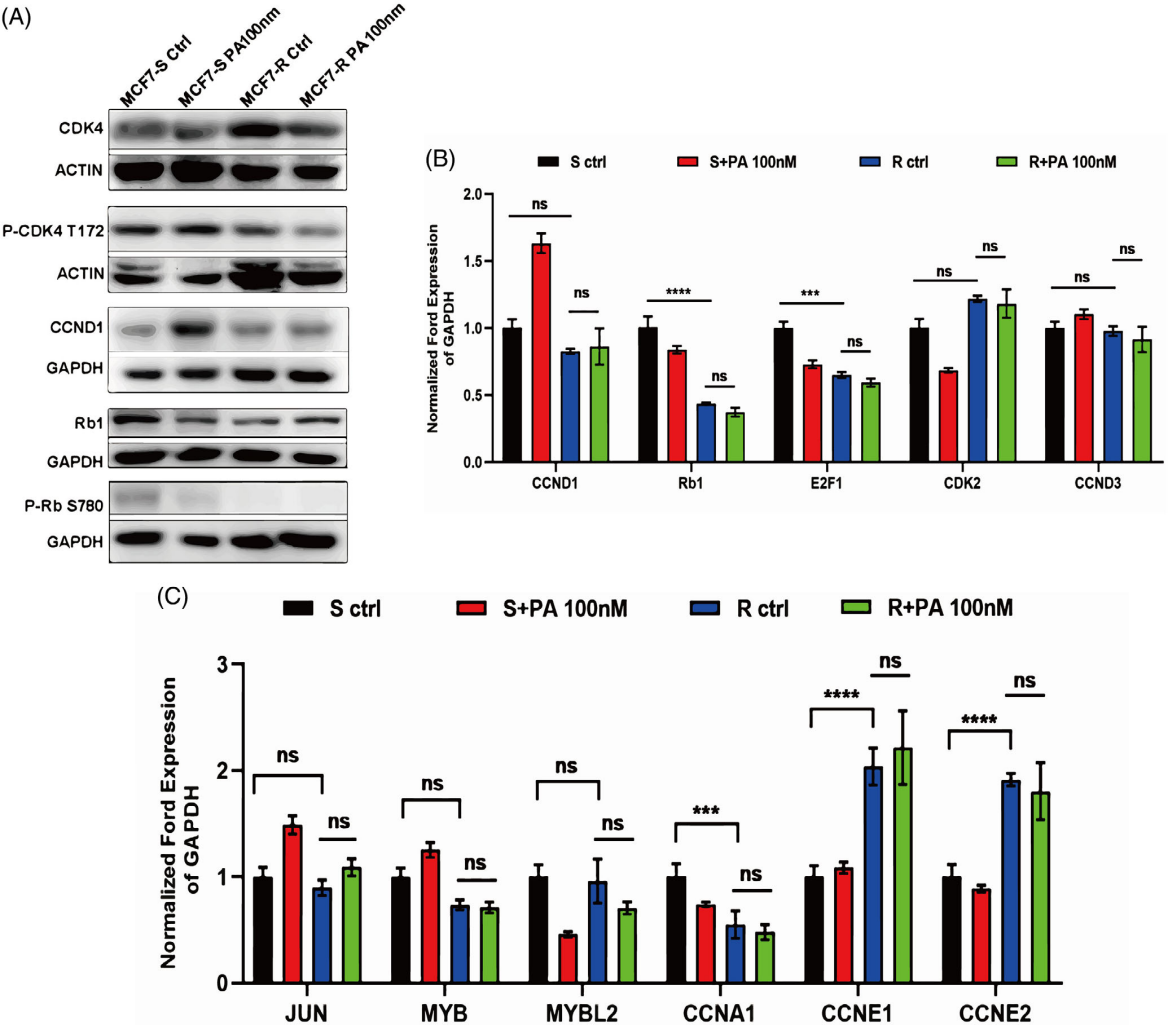
The CDK4/6‐Rb‐E2F1 axis is not altered in MCF‐7/R cell. (A) Immunoblotting analysis of the activation of CDK4 downstream Rb1 signalling pathway in MCF‐7/S and MCF‐7/R cells. (B) RT‐qPCR detection of the expression of CDK4/6‐Rb axis‐related signature genes at the mRNA level in MCF‐7/S and MCF‐7/R cells with or without palbociclib treatment. (C) RT‐qPCR detection of the expression of E2F1 target genes in G1/S phases at the mRNA level. Data represent mean ± SEM. The experiments were repeated at least two times to observe concordant statistical significance. ns, no significance, ***p < 0.001, ****p < 0.0001.

Although the mechanisms of drug resistance are varied, researchers summarize them in the following three convergences: pathway reactivation, pathway bypass and pathway indifference. CDK4 and CDK6 can interact with cyclin D and phosphorylate Rb to promote G1/S transition in HR^+^ BC cells. Palbociclib directly binds to CDK4/6 ATP pocket and suppresses the phosphorylation of Rb by CDK4/6‐cyclin D‐Rb axis. To identify whether the original target pathway, CDK4/6‐Rb pathway, is reactivated after palbociclib resistance, we used a western blotting‐based array to examine the protein expression levels and phosphorylation levels of palbociclib targets in MCF‐7/R cells. Surprisingly, palbociclib targets involved in cell cycle regulation —including CDK4, CCND1 and Rb1—were not significantly altered between MCF‐7/S and MCF‐7/R groups (Figure 2A). In addition to this, Rb1‐S780 phosphorylation was still inhibited by palbociclib, which means that palbociclib resistance is not mediated through reactivation of the CDK4/6‐Rb pathways. Simultaneously, we performed quantitative PCR (qPCR) assays to analyse the mRNA levels of CDK4/6‐Rb axis‐related genes. Consistent with our observations that the CDK4/6‐Rb pathways were not reactivated in MCF‐7/R cells following CDK4/6 inhibitor treatment, we did not observe a significant change in the CDK4/6‐Rb axis‐related genes, CDK2, Rb1, CCND1 and CCNA1 (Figure 2B).

We thus continued to investigate the downstream targets of E2F1 that might be changed in transcriptional activity after palbociclib resistance. From the published literature, we identified some E2F1 target genes that are critical for G1 phase and G1/S phase of the cell cycle. To indicate the transcriptional activity of E2F1 in MCF‐7/S and MCF‐7/R cells, RT‐qPCR experiments of these target genes were conducted. The results showed that the transcriptional activity of almost all E2F1 target genes, as stated above, was essentially unchanged (Figure 2C). Conclusively, we demonstrated that ER+ BC cells acquired palbociclib resistance by activating an alternative signal pathway, independent of the CDK4/6‐PB signal transduction.

### 3.3 The elevated stemness is responsible for palbociclib resistance in MCF‐7 cells

To further explore the mechanism of resistance to palbociclib, we performed transcriptional profiling of MCF‐7/S and MCF‐7/R cells by RNA sequencing (RNA‐seq). In this study, differentially expressed genes (DEGs) of palbociclib treated MCF‐7 cells were compared with *p* < 0.05 and fold change ≥ 2. The gene expression profiles of the treated cells were analysed, and the results revealed that 252 genes were significantly upregulated while 254 genes were significantly downregulated (Figure S3A). Furthermore, the expression patterns were also represented using a volcano plot, which showed that the PA‐tolerant cell line exhibited an altered gene expression pattern. Gene ontology (GO) enrichment analysis revealed that stemness and biosynthesis‐related genes were enriched in the MCF‐7/R group (Figure S4A). L1000CDS2 is a LINCS L1000 characteristic direction signatures search engine.^32^, ^33^ L1000CDS2 predicts and prioritizes a series of small molecules that are predicted to mimic or reverse the input gene expression signatures. Furthermore, we used the L1000CDS2 Query to identify small molecules that reverse the effects of a gene expression signature of MCF‐7/R generated from a differential gene expression analysis. As the results shown in Figure S4B, the score of saracatinib (Src inhibitor) was high.

Src(c‐Src) belongs to the non‐receptor tyrosine kinase family and has been confirmed to participate in cell proliferation, differentiation and invasion.^34^ Additionally, ER‐Src axis was reported to relate to tamoxifen resistance in ER^+^ BC.^35^ In our results, Src was also down‐regulated in MCF‐7/R group. Thus, we found a potential target and further evaluated the treatment effects of saracatinib in vitro. We first chose a concentration gradient (0.1, 0.5, 1 and 5 μM) of saracatinib by literature queries and performed MTT assays to determine cell viability after saracatinib treatment. The results indicated that MCF‐7/R cells were resistant to saracatinib and that single‐drug treatments were poorly effective (Figure S4C). Therefore, the combination of saracatinib plus palbociclib was needed to treat drug resistance. MTT assays demonstrated that the efficacy of the combination therapy in killing MCF‐7/R cells in vitro was superior to that of the single‐drug treatment (Figure S4D). Subsequently, we carried out cell cycle experiments to further evaluate the effect of the combination treatment therapy on cell cycle arrest. The results shown in Figure S4G indicated that the combination of saracatinib and palbociclib enabled MCF‐7/R cells to partially regain sensitivity to palbociclib, and its therapeutic effects were superior to palbociclib monotherapy.

As described above, saracatinib exhibited cytotoxic activity against MCF‐7/R cells to some extent and palbociclib did not cause apoptosis in MCF‐7/S or MCF‐7/R cells, even at a high concentration of 10 μM (Figure S4E,F). Thus, we wanted to know whether a single saracatinib treatment or the combination of saracatinib plus palbociclib could cause cell apoptosis in MCF‐7/S and MCF‐7/R cells. Given this, double staining of cells with Annexin‐V/PI was performed to determine the effect of saracatinib on apoptosis. In MCF‐7/S cells, saracatinib alone or saracatinib plus palbociclib could not induce cell apoptosis (Figure S4E,F). Meanwhile, MCF‐7/R cells treated with saracatinib alone showed similar results. However, the percentage of Annexin‐V^+^/PI^+^ (double positive) apoptotic cells increased by approximately 7% in the saracatinib and 100 nM palbociclib group. The apoptotic effect was increased (~20%) with increasing palbociclib concentrations (1 μM palbociclib combined with saracatinib). Collectively, these results offer new directions for solving outstanding problems in drug resistance.

Considering the different characteristics of MCF‐7/S and MCF‐7/R cells, we focused on the differential analysis of stemness, cell cycle and cancer aggressiveness‐related genes in these two cell lines. Notably, MCF‐7/R cells presented a different transcriptomic landscape as compared to normal MCF‐7 cell lines, particularly in terms of cell stemness and invasion (Figure S4A). Concurrently, a mammosphere formation assay was performed as an auxiliary method to assess the difference in cell stemness between the MCF‐7/S and MCF‐7/R groups. As shown in Figure S3B, mammosphere formation was strikingly evoked in MCF‐7/R cells, which is consistent with the bioinformatics analysis discussed above.

Several studies have demonstrated that cancer stem cells contribute to chemotherapy resistance and cause treatment failure. As we know, JAK‐STAT3 activation promotes cell stemness and favours the generation of CSCs.^36^, ^37^ Several preclinical studies have also demonstrated that blocking STAT3 signalling with stattic (STAT3 inhibitor) may reduce the stemness of BC cells.^38^, ^39^ To regulate stemness, we used stattic to block STAT3 activation in MCF‐7/R cells. To determine whether stattic regulates MCF‐7/R cell stemness, the stemness marker CD44v was then measured. Western blot analysis confirmed that the stemness of MCF‐7/R cells was inhibited by stattic (Figure S3D). Then, to examine the relationship between stemness and drug resistance, flow cytometry was performed to test whether the inhibitory effects on the stemness of MCF‐7/R cells exacerbated cell cycle arrest. Additionally, G1/S cell cycle arrest occurred in MCF‐7/R cells treated with palbociclib and stattic (Figure S3C). The results indicated that palbociclib resistance is reversible and that the inhibition of stemness can effectively restore the susceptibility of MCF‐7/R cells to palbociclib. In summary, we concluded that drug resistance in MCF‐7/R cells was mediated, at least in part, by the upregulation of cell stemness.

### 3.4 Palbociclib induces cellular senescence and senescence‐associated reprogramming turns senescent cell into a stem‐like state

**FIGURE 3:**
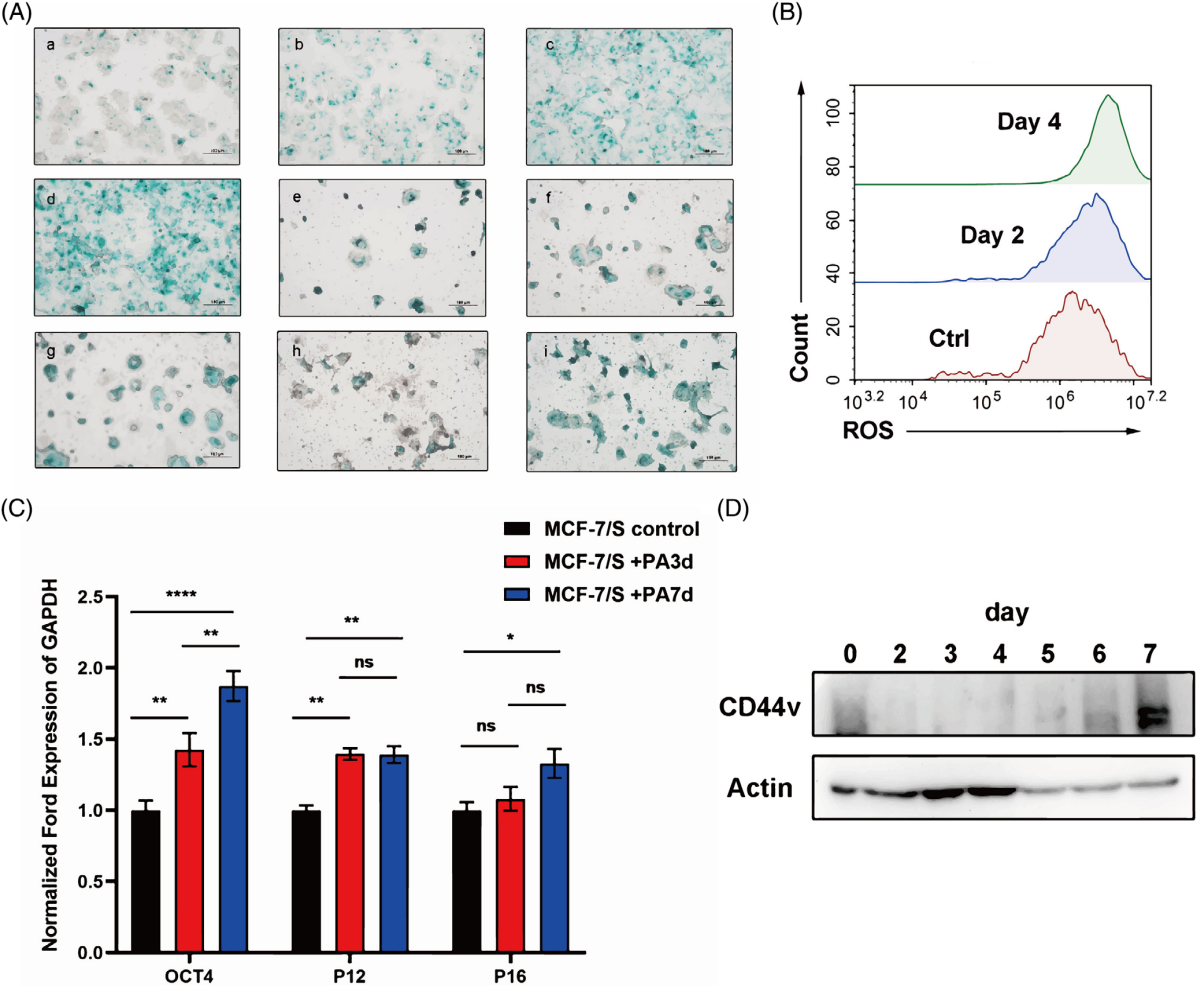
Palbociclib treatment evoked cellular senescence of ER^+^ BC cells, and then the senescence‐like phenotype promoted stemness of ER^+^ BC cells. (A) SA‐β‐Gal staining of MCF‐7/S cell after treatment of palbociclib. From the fourth day after palbociclib treatment, the cell senescence was monitored every 2 days for 20 days. a: Day 4; b: Day 6; c: Day 8; d: Day 10; e: Day 12; f: Day 14; g: Day 16; h: Day 18; i: Day 20 after palbociclib treatment. Scale bars, 100 μm. (B) The ROS levels were assessed by flow cytometer after palbociclib exposure to MCF‐7/S cells and stained with DCFH‐DA. (C) RT‐qPCR detection of the expression of stemness and senescence‐related genes in MCF‐7/S cells after palbociclib treatment at the mRNA level. (D) Immunoblotting analysis of the CSC marker, CD44v, expression in MCF‐7/S cells with palbociclib treatment.

Preclinical studies have shown that cell cycle arrest caused by CDK4/6 inhibition can elicit cellular senescence in multiple tumour types.^40^ A well‐recognized characteristic of CDK4/6 inhibitor treatment is the induction of senescence. To assess whether palbociclib could lead to cellular senescence, we observed the expression of senescence‐associated β‐galactosidase (SA‐β‐gal) by β‐gal staining assay. As mentioned above, MCF‐7 cells showed a larger positive stain, a marker of significant cellular senescence, 3 days after palbociclib treatment (Figure 3A). Recent studies showed that increased ROS production is associated with cellular senescence. Moreover, we noted that palbociclib boosted ROS in a time‐dependent manner (Figure 3B). Concurrently, the markers of senescence, including P12 and P16, were upregulated during palbociclib treatment for 3 days. Cellular senescence and tumour cell dormancy are important concepts in cancer therapy. Recent studies have shown that cellular senescence is a major contributor to the increase in CSCs.^41^ When EGFRvIII expression was blocked in established tumours by treating the mice with Dox, the tumours entered a temporary state of dormancy and then, released from dormancy, re‐establishing aggressive growth. In fact, tumour dormancy and senescence or stemness are not mutually exclusive. It is conceivable that cancer cells in a senescence‐like state might remain ‘dormant’ tumour cells and therefore represent a dangerous potential for tumour relapse. Therefore, we examined the expression of CD44v in MCF‐7 cells during palbociclib‐induced senescence. MCF‐7 cells acquired enhanced stemness‐related properties after undergoing cellular senescence (Figure 3D). The mRNA level of OCT4, a marker of stemness, was upregulated and this was consistent with the western blot analysis above. Based on this, we speculate that the process of chemotherapy‐induced senescence promotes cancer stemness.

### 3.5 PFKFB4 is essential for senescence‐associated stemness transformation

**FIGURE 4:**
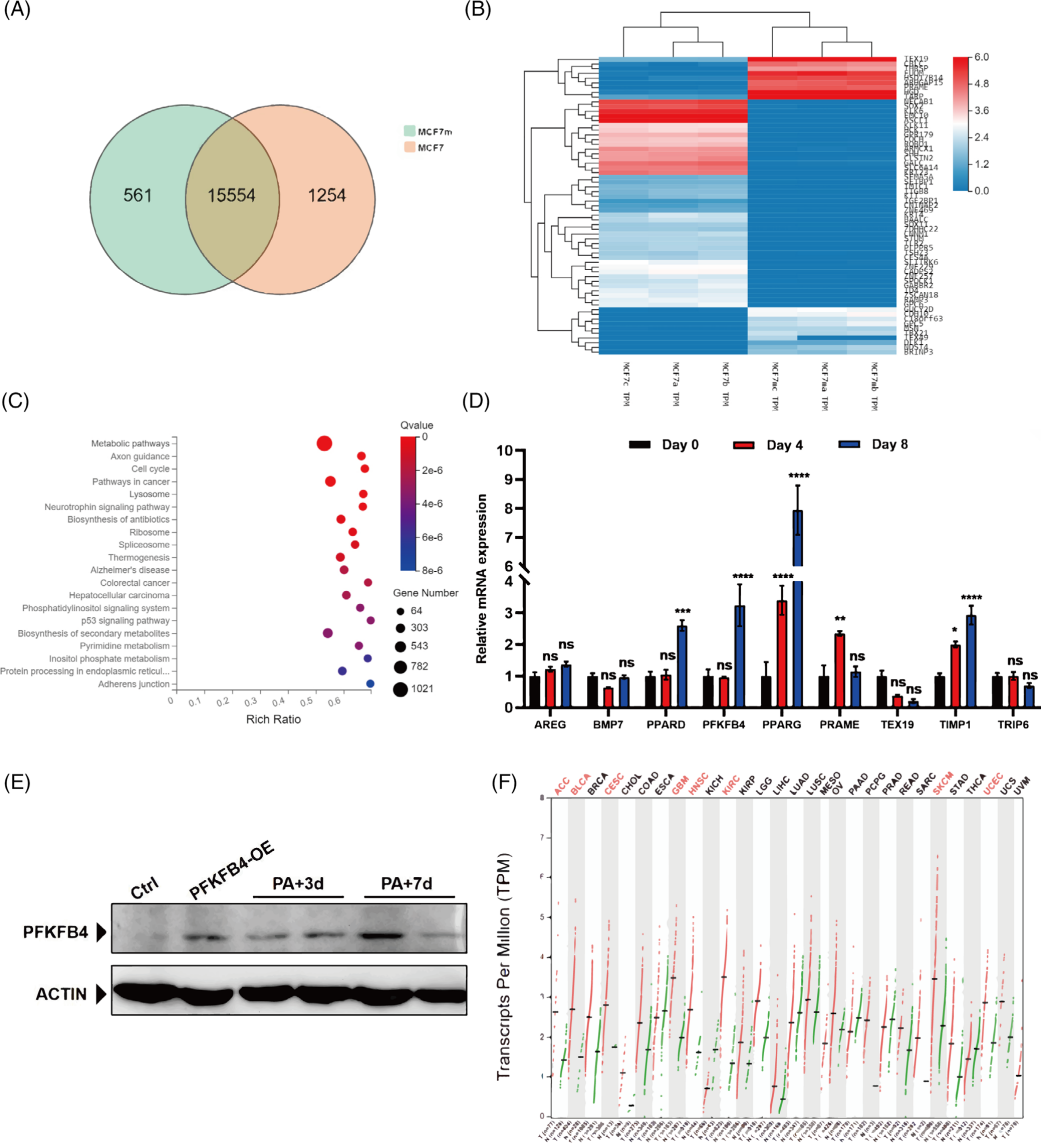
RNA‐sequencing identified the key molecule during drug resistance to palbociclib. (A) Venn diagram of the numbers of DE genes from MCF‐7/S versus MCF‐7/m (7 days after palbociclib treatment). (B) Heatmap of log2 fold changes of overlapping genes (from the Venn diagram intersection). (C) GO enrichment analysis of differentially expressed genes between MCF‐7/S and MCF‐7/m cells. (D) Validation of the RNA‐sequencing results by RT‐qPCR. Data represent mean ± SD. The experiments were repeated at least two times to observe concordant statistical significance. (E) Immunoblotting analysis of PFKFB4 expression in MCF‐7/S cells with palbociclib treatment. (F) Comparison of PFKFB4 expression between tumour and normal samples. Tumour types with a significant difference (*q* value <0.01) between tumour and normal samples are marked with red colour. ns, no significance, *p < 0.05, **p < 0.01, ***p < 0.001, ****p < 0.0001.

Given the findings above, we monitored changes in the stemness of MCF‐7 cells after palbociclib treatment in real time. At Day 7, the stemness was obviously elevated (Figure 3D). Then, RNA sequencing was performed for comparative gene expression profiling of these cells (we named them ‘MCF‐7m’). Collectively, compared with normal MCF‐7 cells, MCF‐7m cells exhibited unique transcriptome landscapes. A total of 1815 genes were identified as differentially expressed genes between the MCF‐7 and MCF‐7 m groups (Figure 4A,B). KEGG pathway enrichment analysis showed that these DEGs were significantly enriched in many cancer‐related pathways, such as metabolic pathways, the cell cycle signalling pathway, and the P53 signalling pathway (Figure 4C). According to the literature search and the accumulated experience, we selected several candidate genes that may play an important role in stemness transformation. Validation of selected differentially expressed genes was performed by qPCR analysis. As expected, the results obtained from qPCR and RNAseq were highly comparable, especially PFKFB4 (Figure 4D).

Next, we performed western blot assays to analyse the changes in PFKFB4 protein expression during palbociclib treatment. The results showed that the expression of PFKFB4 is increased in the process of palbociclib treatment (Figure 4E). As expected, this conclusion was consistent with the RNAseq and q‐PCR results. The 29 tumour types with more than 9727 cases in TCGA database were analysed. Consistent with our findings above, PFKFB4 expression was upregulated in multiple tumours compared with paracarcinoma tissues (Figure 4F). Therefore, we considered that PFKFB4 plays a key role in tumour development during palbociclib treatment.

### 3.6 Increased PFKFB4 level is associated with cellular senescence and PFKFB4 promotes cell stemness in BC cell

**FIGURE 5:**
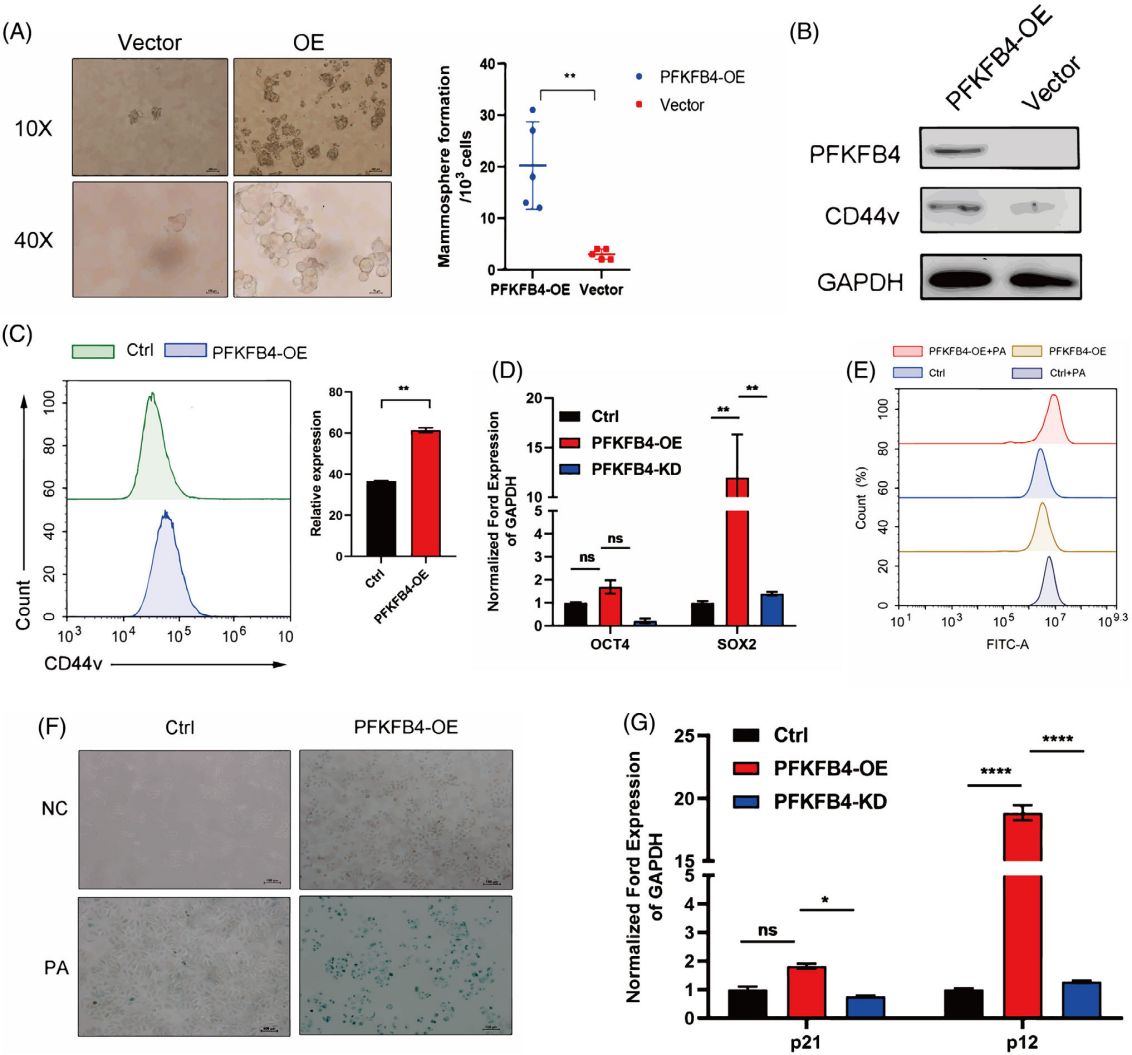
Increased PFKFB4 level was associated with cellular senescence and PFKFB4 promoted cell stemness in ER^+^ breast cancer cell. (A) Overexpression of PFKFB4 in MCF‐7/S cell (PFKFB4‐OE MCF‐7/S). MFA detected self‐renewal capacity. (B) Immunoblotting analysis of PFKFB4 and CD44v expression in PFKFB4‐OE MCF‐7/S cells. (C) The stemness of PFKFB4‐OE MCF‐7/S cells was analysed by flow cytometry. (D) SA‐β‐Gal staining of MCF‐7/S and PFKFB4‐OE MCF‐7/S cells after treatment with palbociclib. (E) The ROS levels were assessed by flow cytometer after palbociclib exposure to MCF‐7/S and PFKFB4‐OE MCF‐7/S cells and stained with DCFH‐DA. (F) Detecting the expression of stemness and (G) senescence‐related markers in normal, PFKFB4‐OE and PFKFB4‐knockdown MCF‐7/S cells. Data represent mean ± SEM. The experiments were repeated at least two times to observe concordant statistical significance. ns, no significance, *p < 0.05, **p < 0.01, ***p < 0.001.

To investigate the exact role of PFKFB4 in stemness transformation, we performed a series of in vitro experiments. First, we constructed an MCF‐7 cell line with stable overexpression of PFKFB4. Then, a mammosphere formation assay (MFA), an in‐vitro surrogate for stemness, was performed to evaluate the self‐renewal capacity of the PFKFB4‐overexpression cell line. As shown in Figure 5A, the PFKFB4‐overexpression MCF‐7 group significantly promoted mammosphere formation. The transmembrane glycoprotein CD44 is a representative molecular marker for BC stem cells and regulates the adaptive plasticity of tumour cells. Accumulating evidence has demonstrated that CD44 alternative splicing frequently occurs during cancer progression. Recently, several studies indicated that CD44 variant (CD44v) isoforms are more accurate CSC markers than CD44 standard (CD44s).^42^, ^43^ As mentioned above, western blot analysis indicated that CD44v was more highly expressed in PFKFB4‐overexpression MCF‐7 cells than in MCF‐7 cells transduced with the control vector (Figure 5B). Given that CD44v is a cell surface protein, we further detected the expression of CD44v in PFKFB4‐OE and normal MCF‐7 cells by flow cytometry. Results from the flow cytometry analysis showed that CD44v was overexpression at the surface of PFKFB4‐OE cells, which is consistent with those obtained from the western blot assay (Figure 5C). Moreover, cancer cell stemness markers OCT4 and SOX2 were detected quantitatively and high levels of SOX2 were found in PFKFB4‐OE MCF‐7 cells compared with normal MCF‐7 cells (Figure 5F). These observations support well that PFKFB4 could promote the stemness of MCF‐7 cells.

Meanwhile, MCF‐7 cells appeared to undergo chemotherapy‐induced senescence during palbociclib treatment. This (process) was accompanied by elevated PFKFB4 expression. Notably, a senescence‐associated β‐galactosidase (SA‐β‐gal) staining assay indicated that the forced expression of PFKFB4 in MCF‐7 cells strongly exacerbated senescence during palbociclib treatment (Figure 5D). Recently, several studies have reported that senescent cells secrete a set of molecules (senescence‐associated secretory phenotype, SASP).^44^ Furthermore, SASP expression causes ER stress and in turn activates reactive oxygen species (ROS) production. Hence, we measured the levels of ROS in PFKFB4‐OE and normal MCF‐7 cells treated with palbociclib using DCFH‐DA. In line with our reasoning, the ROS level was significantly increased in PFKFB4‐OE MCF‐7 cells upon palbociclib treatment compared with normal MCF‐7 cells (Figure 5E). In addition, the markers of cell senescence, including P12 and P21, were also upregulated in PFKFB4‐OE MCF‐7 cells. Thus, we hypothesized that PFKFB4 could play an important role in favouring senescence‐associated stemness.

### 3.7 PFKFB4 promotes cell stemness by enhancing glycolysis and evokes metabolic reprogramming

**FIGURE 6:**
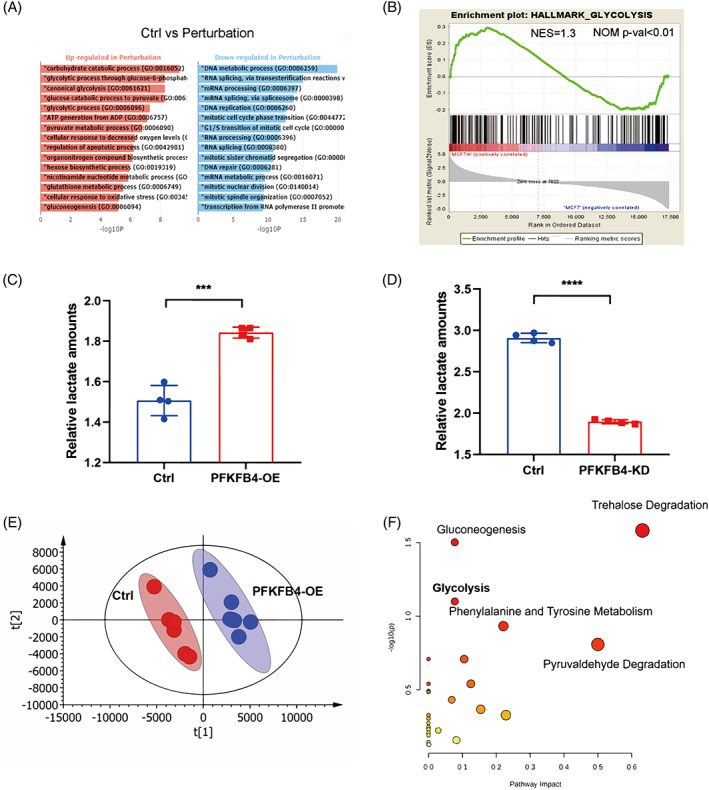
PFKFB4 promoted cell stemness by enhancing glycolysis in ER^+^ breast cancer cell. (A) Gene ontology enrichment analysis results. The *x*‐axis indicates the −log10(*p* value) for each term. Ctrl: MCF‐7/S cell; Perturbation: MCF‐7/m. (B) Gene set enrichment analysis of alterations in Glycolysis signature genes after palbociclib treatment for 7 days. (C) Measurement of lactate secretion for MCF‐7/S cells with control and PFKFB4 overexpression (PFKFB4‐OE). (D) Measurement of lactate secretion for MCF‐7/S cells with control and PFKFB4 knockdown (PFKFB4‐KD). (E) PLS‐DA score plot in MCF‐7/S (Ctrl) cell supernatant group and PFKFB4‐OE MCF‐7/S (PFKFB4‐OE) cell supernatant group detected in positive ion mode. (F) Pathway analysis of the differential metabolites between MCF‐7/S (Ctrl) cell supernatant group and PFKFB4‐OE MCF‐7/S (PFKFB4‐OE) cell supernatant group. Data represent mean ± SEM. *p < 0.05, **p < 0.01, ***p < 0.001, ****p < 0.0001.

Then, we explored how PFKFB4 participated in the promotion of BC stemness. To solve this question, GO enrichment analysis was conducted. We found that several pathways were significantly enriched in MCF‐7m cells, such as the canonical glycolysis pathway, pyruvate metabolic process and glycolytic process (Figure 6A). Notably, these pathways were mostly involved in the glycolysis metabolic pathway. Meanwhile, Gene set enrichment analysis (GSEA) revealed that a previously established glycolytic metabolic signature was strongly skewed towards the palbociclib‐treated group (Figure 6B). Considering that PFKFB4 is a key bifunctional enzyme in glycolysis, we speculated that palbociclib promotes cancer stemness via PFKFB4‐mediated glycolysis.

First, we evaluated whether PFKFB4 was able to regulate the glycolysis process. The major characteristics of aerobic glycolysis are elevated glucose uptake and lactic acid secretion. Overexpression of PFKFB4 was able to significantly increase lactic acid secretion (Figure 6C). Similarly, deletion of PFKFB4 could reduce the metabolic secretion of lactic acid (Figure 6D). To comprehensively characterize the effect of PFKFB4 on cell metabolism, we then performed untargeted metabolomics profiling of PFKFB4‐OE MCF‐7 cells and cell supernatants by HPLC/Q‐TOFMS in positive ion mode.

To validate the LC–MS method, we first performed the alignment and normalization of the QC sample data. Next, the detected ions were then cleaned by the 80% rule in order to better assess the reproducibility of the developed method. As an obvious result, nearly 85% of the detected ions with an RSD value (relative standard deviation) in the cell supernatant group were less than 30% in positive ion mode and more than 85% in the cell group (Figure S5A,D). The above results revealed that the developed method has good reproducibility and robustness and can be applied for the following study. Afterwards, we analysed the retention time, *m/z* and peak areas from various perspectives by SIMCA‐P software. Partial least squares‐discriminant analysis (PLS‐DA) was implemented to illustrate the intervention effects of PFKFB4. As shown in Figure 6E, distinct clustering of the PFKFB4‐OE and CON groups was observed at both the cell and cell supernatant levels. This segregation between the PFKFB4‐OE and CON groups suggested that PFKFB4 evokes certain significant biochemical changes and alters the metabolic status of BC cells.

Accordingly, we performed orthogonal partial least squares‐discriminant analysis (OPLS‐DA) to explore the differences between the PFKFB4‐OE group and the CON group. From Figure S5B,E, we observed that the cell lysate and cell supernatant samples were clearly separated into two blocks by the score plot of OPLS‐DA in positive ion modes, which indicates that the PFKFB4‐OE group and CON group share different metabolic profiles and PFKFB4 may lead to metabolic reprogramming. Then, the corresponding S‐plot was applied to identify the differential metabolites for discrimination. According to VIP values (exceeding 2.0), we selected a total of 18 differential metabolites in cell supernatant samples as potential biomarkers and 24 in cell lysate samples between the PFKFB4‐OE group and the CON group (Figure S5C).

The metabolites were identified by the human metabolome database (HMDB). The corresponding chemical information of these compounds is shown in Tables S1 and S2. These biomarker candidates suggested that the metabolic pathways were affected by PFKFB4. Considering the significant differences, we used the MetaboAnalysis website to investigate relevant metabolic pathways. The affected pathways are depicted in Figure 6F. Among them, enhanced glycolysis and pyruvate metabolism were considered as the most likely causes of the effects of PFKFB4 on BC cells.

In addition, purine metabolism was an enriched pathway according to the pathway enrichment analysis in metabolomics profiling of cell lysates (Figure S5F). Recently, purine metabolism has been reported to be associated with the cell cycle and signal transduction. This result suggested that PFKFB4 plays a role in cell‐cycle disorders and cancer progression.

In summary, the above results showed that glycolysis‐related secretions were upregulated in PFKFB4‐OE MCF‐7 cell supernatants. Pathway analysis identified the glycolysis pathway as an enriched pathway in the PFKFB4‐OE group. Moreover, PFKFB4 could affect the metabolism of MCF‐7 cells. Altogether, PFKFB4 caused metabolic reprogramming of tumour cells and enhanced glycolysis.

We previously screened and identified KAN0438757 (henceforward, KAN) as a PFKFB4 selective inhibitor.^45^ KAN successfully attenuated PFKFB4 expression (Figure S6A). Moreover, treatment with 30 μM KAN for 24 h significantly decreased glucose consumption (Figure S6B.a). In addition, even 10 μM KAN reduced glucose uptake in MCF‐7 cells after 48 h of drug treatment (Figure S6B.b). In brief, the PFKFB4 inhibitor is able to inhibit glycolysis. We subsequently verified the connection between glycolysis and stemness. The decreased glucose consumption and lactate secretion suggested that increased glycolysis in palbociclib‐treated PFKFB4‐OE MCF‐7 cells can be suppressed by the glycolysis inhibitor IAA^46^ (Figure S6C,D). Interestingly, PFKFB4‐OE MCF‐7 cell stemness transformation was repressed upon glycolysis inhibition (Figure S6E). This further illustrates that BC stemness is regulated by PFKFB4‐mediated glycolysis.

### 3.8 Intervention of PFKFB4 influences the BC cell drug sensitivity to palbociclib

**FIGURE 7:**
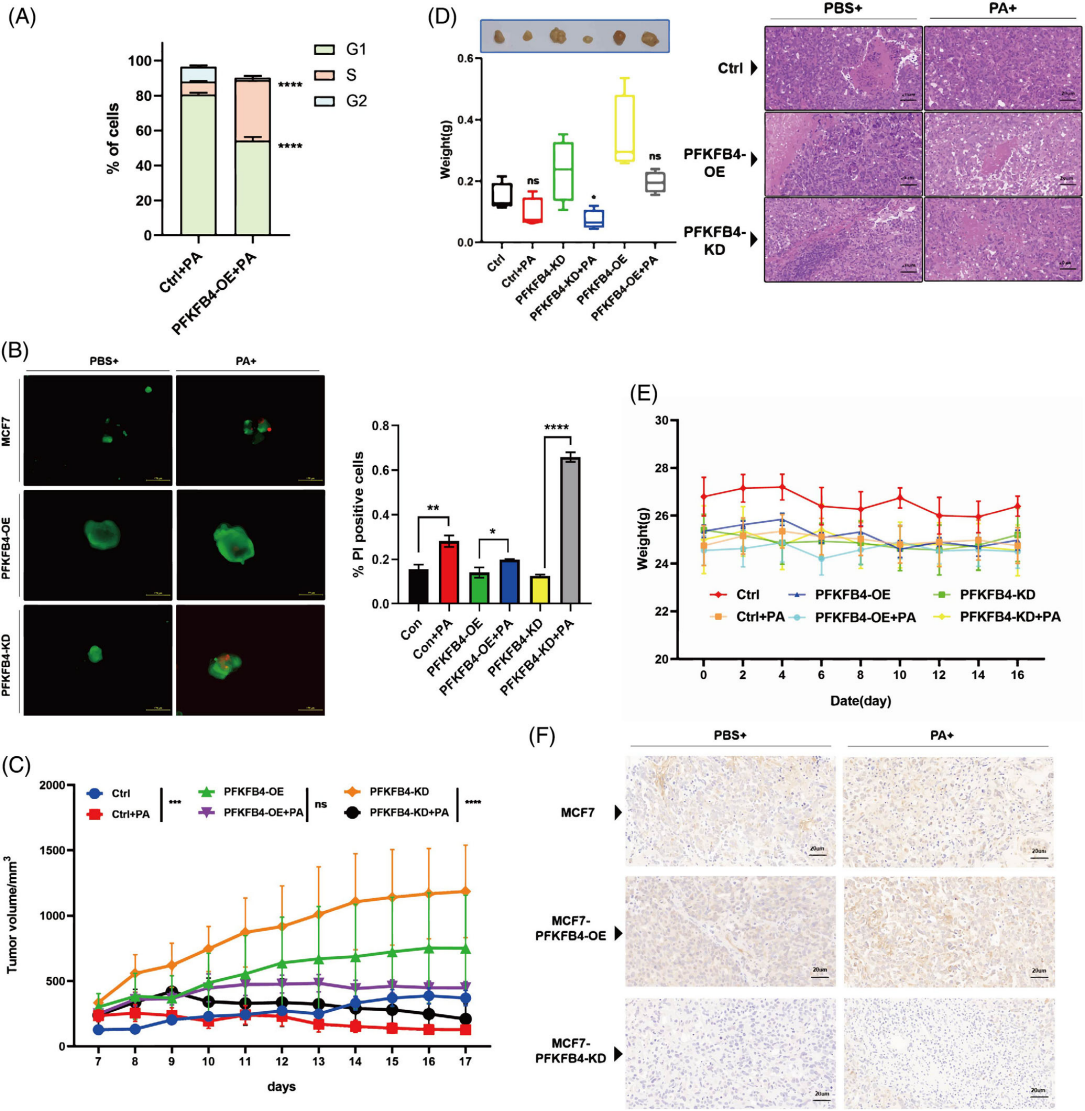
Intervention of PFKFB4 influences the breast cancer cell drug sensitivity to palbociclib. (A) The percentage of cells in G1, S and G2 phase stages of the cell cycle was quantified in MCF‐7/S and PFKFB4‐OE MCF‐7/S cells with palbociclib treatment. (B) The tumorsphere killing assay in Ctrl, Ctrl + PA, OE, OE + PA, KD and KD + PA groups, green represents live cells (Calcein AM); red represents dead cells (PI). Scale bars, 100 μm. Intervention of PFKFB4 influences the effectiveness of palbociclib in the inhibition of tumour growth and weight in vivo. (C) Tumour growth curves of tumour‐harbouring mice treated with PBS or palbociclib (100 mg/kg) for every day. (D) Representative tumour pictures (top panel) and tumour weights (bottom panel).The haematoxylin and eosin (H&E) staining of tumour tissue, Scale bars, 20 μm. (E) Body weights of tumour‐harbouring mice in each group. (F) Representative IHC pictures of ALDH1 in tumours with indicated treatments in each group. Scale bars, 20 μm. Data represent mean ± SD. *n* = 6. ***p < 0.001, ****p < 0.0001.

To explore the role of PFKFB4 in drug resistance, the cell cycle of PFKFB4‐OE MCF‐7 cells after palbociclib treatment for 48 h was tested by flow cytometry. The results showed that forced PFKFB4 overexpression abrogated G1/S phase cell cycle arrest, which was induced by palbociclib (Figure 7A). Therefore, our finding that PFKFB4 is upregulated in PA‐treated MCF‐7 cells and that overexpression of PFKFB4 promotes drug resistance in MCF‐7 cells implicates PFKFB4 as an attractive anti‐resistance target.

As we all know, 3D tumour spheres provide an improved artificial model that closely mimics the biological properties of tumours in vivo compared with the conventional 2D culture method.^47^ 3D tumour spheres from in vitro cultures are an effective means to evaluate the efficacy of anticancer drugs. We made use of MCF‐7 cell‐derived spheres, PFKFB4‐OE‐MCF‐7 spheres and PFKFB4‐KD‐MCF‐7 spheres to assess the role of PFKFB4 in palbociclib resistance by calcein AM/PI staining. When treating the spheres with 1 μM palbociclib, the viability of the PFKFB4‐OE‐MCF‐7 group was not significantly affected (Figure 7B). The other two groups showed strong sensitivity to palbociclib. Notably, the PFKFB4‐KD‐MCF‐7 group displayed the greatest sensitivity among them.

To analyse the ability of PFKFB4 to reverse drug resistance, we established three BC xenograft models by subcutaneously implanting normal MCF‐7 cells and genetically modifying MCF‐7 cells, overexpressing or knocking down PFKFB4, into immunodeficient mice. After 1 week, tumour‐harbouring mice were divided into six groups, including three untreated control groups and three palbociclib treatment groups (i.e., Ctrl, Ctrl+PA, OE, OE + PA, KD and KD + PA groups). Tumour volumes and body weights were monitored daily for up to 17 days until the mice were euthanized. Tumour growth curves indicated that PFKFB4 enhanced the drug resistance of BC cells to palbociclib (Figure 7C). Likewise, the knockdown of PFKFB4 remarkably improved drug sensitivity and enhanced the response of BC cells to palbociclib, which led to decreased tumour volume and weight (Figure 7D). Notably, none of the mice showed the obvious difference in body weight or died in any of the six groups during palbociclib treatment, suggesting minimal toxicities (Figure 7E). Moreover, the qPCR and IHC results indicated that the knockdown of PFKFB4 could decrease the cancer stemness and EMT capability, which was evidenced by reduced levels of stemness and EMT‐related markers (Figures 7F and S7A,B).

Conclusively, we confirmed that intervention of PFKFB4 could indeed modify the sensitivity of ER^+^ BC cells to palbociclib in vivo.

## 4 DISCUSSION

Our current study provided novel insights into the mechanisms underlying ER^+^ BC drug resistance and demonstrated that PFKFB4 plays an essential role during tumour cell stemness transformation and acquired CDK4/6 inhibitor resistance.

In the CDK family, CDK4 and CDK6 are the most famous members due to their fundamental roles in driving the cell cycle and regulating carcinogenesis and progression in BC. Actually, several studies have demonstrated that the proliferation of ER^+^ BC is especially dependent upon CDK4 and CDK6, which promote G1 to S phase progression. Given this, a series of selective oral CDK4/6 inhibitors, such as palbociclib, have been developed for the treatment of ER^+^ breast tumours. However, Pfizer funded the PALOMA‐2 clinical trial and randomly assigned 666 women with ER(+)/HER2(−) advanced BC in 17 countries from February 2013 to July 2014.^13^ Among these patients, more than 35% experienced disease progression and cancer recurrence during CDK4/6 inhibitor treatment for 2 years. The possible mechanisms include the elevated activity of the target such as CDK6 and overactivation of downstream kinases, for example, CDK2. In the current study, we developed palbociclib‐resistant ER^+^ BC cell line to interrogate the drug resistance mechanism. By using multiple experimental approaches, we found that palbociclib has the potential to act independently of the CDK4/6‐cyclin D‐RB pathway, likely due to the acquired CCNE1 alterations. In fact, CDK2/CCNE1 complex phosphorylates Rb1, thereby leading to the loss of Rb1 activity.^48^ Then, as an E2F1 partner, phospho‐Rb1 promotes dissociation of the Rb/E2F1 complex and activation of E2F1. Coincidentally, CCNE1 is a downstream target gene of E2F1.^49^ Therefore, CCNE1 and E2F1 form a feedback loop and hyperphosphorylate Rb1 to achieve CDK4/6i resistance. To determine whether CCNE1 plays a key role in palbociclib resistance, we utilized siRNA knockdown of CCNE1 and detected cell cycle changes in MCF‐7/R cells. Moreover, silencing of CCNE1 alone in MCF‐7/R cells had little effect on cell‐cycle arrest, but led to increased cell‐cycle arrest in combination with palbociclib. The experimental results showed that CCNE1 knockdown can partially restore sensitivity to palbociclib in MCF‐7/R cells and bring us new inspiration that targeting CCNE1 may sensitize ER^+^ BC to palbociclib therapy (Figure S8).

There is a consensus recognition that CDK4/6 inhibition as monotherapy for ER^+^ BC is restricted to early adaptative response, with the complex of cyclin D1 and CDK2 mediating residual cell‐cycle entry.^6^ This underscores that combining CDK4/6 inhibitors with other therapies, including regulation of cyclin D1 and other G1/S cyclins, inhibition of PI3K or mTORC1, opens a new path to develop novel strategies treating ER^+^ BC.^6^, ^50^, ^51^ Notably, although early palbociclib and PI3K inhibitor GDC‐0941 combinations failed to fully resensitize palbociclib‐resistant cell lines, this treatment strategy prevented the acquisition of resistance. The laboratory finding suggests that instead of targeting and reversing the acquisition of resistance, we can seek to propose a novel strategy to block early adaption and prevent/delay drug resistance. Inspired by this idea, we monitored the phenotype changes of the ER^+^ BC cell treated with palbociclib to explore the possible factor driving palbociclib resistance. Interestingly, continuing treatment of palbociclib evoked cellular senescence of ER^+^ BC cells, and then, the senescence‐like phenotype promoted stemness of ER^+^ BC cells, accompanied by increased chemoresistance and tumour‐initiating potential. Cellular senescence is a cell‐cycle arrest program in response to intrinsic and/or extrinsic stress and seems to be incompatible with stemness, which represents a stem cell‐like state.^44^ Nevertheless, as research on cellular senescence indicated, senescence might not reflect a terminal state of restricted cell expansion. Some senescence‐like cells appear to escape spontaneous cell death, recover with self‐renewal capacity and form stable colonies with aggressive cancer stem‐like cell activity.^41^ However, the specific mechanism that mediates the senescence–stemness alliance remains unknown. By applying a transcriptome analysis, we first identified PFKFB4 as the key regulatory node in senescence‐associated stemness transformation in ER^+^ BC cells during palbociclib treatment.

Recent studies have revealed that energy metabolism reprogramming enables stemness programs.^52^ Metabolic reprogramming is a hallmark of cancer and tumour cells redirect energy metabolism towards glycolysis for survival and proliferation, especially in a hypoxic microenvironment. Glycolysis supports multiple regulatory pathways via enhanced pyruvate oxidation, which may alter stemness‐related signalling networks. In addition, it has previously been reported that glycolysis promotes acquired stemness phenotype in pancreatic cancer cells and nasopharyngeal carcinoma cells.^53^, ^54^ Hence, we speculate that glucose metabolism reprogramming may play an important role in stemness transformation in ER^+^ BC. To confirm the hypothesis, we performed untargeted metabolomics to analyse the metabolic status of PFKFB4‐OE ER+ BC cells. As expected, altering glucose metabolism profoundly regulated numerous metabolic pathways. Moreover, we found that metabolism reprogramming is involved in stemness transformation and chemosensitivity alteration. In conclusion, we established a link between metabolism reprogramming and stemness transformation. Meantime, our results provide indirect proof that a combination of glycolysis inhibition with chemotherapy might promote chemosensitivity and prevent chemoresistance.

PFKFB4 can regulate the critical rate‐limiting enzyme in the glycolysis pathway and mediate glucose metabolism. The elevated expression of PFKFB4 effectively promotes glucose uptake and lactic acid secretion, which suggests the occurrence of glucose metabolism reprogramming. In addition, previous studies have shown that PFKFB4 is activated in hypoxia and overexpressed in various types of tumour tissues, especially in metastatic and recurrent malignant tumours.^22^, ^24^, ^55^, ^56^, ^57^ Hence, we speculate that PFKFB4 plays a prominent role in tumour cell stemness transformation and acquired CDK4/6 inhibitor resistance and targeting PFKFB4 might be an ideal strategy for ER^+^ BC chemotherapy. To confirm this hypothesis, we established and comprehensively verified the therapeutic effects of combinatorial treatments with CDK4/6 inhibition and PFKFB4 gene edition in the MCF‐7/PFKFB4‐OE and MCF‐7/PFKFB4‐KD xenograft mice model. Our results suggest that the combination of palbociclib and the depletion of PFKFB4 improves drug sensitivity to palbociclib and is continually maintained in the presence of drugs. Given the results presented in this paper, we considered combining palbociclib with a PFKFB4‐specific inhibitor for subsequent ER^+^ BC therapies.

## 5 CONCLUSIONS

Taken together, our current study uncovered the key role of PFKFB4 in promoting stemness transformation and drug resistance of ER^+^ BC cells under palbociclib treatment. Considering the specific mechanism, PFKFB4 was unearthed to enhance glycolysis and induce glucose metabolism reprogramming. One step further, enhanced glycolysis was proven to fuel self‐renewal and stemness transformation. Eventually, this led to drug resistance to palbociclib and malignant progression. Additionally, PFKFB4 knockdown restored drug sensitivity and demonstrated a potential therapeutic efficacy. Thus, PFKFB4 has good potential to be a therapeutic target to overcome drug resistance to palbociclib in ER^+^ BC and more efforts are needed to develop safe and efficient PFKFB4‐targeted drug and undertake clinical translation in the future.

## AUTHOR CONTRIBUTIONS

Pingping Shen, Sijie Wang and Yuncheng Bei conceived and designed the project. Congying Xie, as a participant, conceived and discussed the project. Sijie Wang, Qiang Tian, Yuncheng Bei, Rui Wang and Qiuping Wang performed the experiments. Sijie Wang, Qiang Tian, Rui Wang and Luchen Sun analysed the data. Sijie Wang and Yuncheng Bei wrote the manuscript. Jiangqiong Ke constructed the palbociclib‐resistant cell line and provide critical clinical proof. Pingping Shen financially supported the study. Pingping Shen supervised the study. All authors read this article and approved the final manuscript.

## CONFLICT OF INTEREST

The authors declare no conflict of interest.

## Supporting information


**FIGURE S1** Construction and evaluation of drug‐resistance ER^+^ breast cancer cell model

**FIGURE S2** Palbociclib caused no change in the expression of PD‐L1 and apoptosis levels in both MCF‐7/R and normal MCF‐7/S cells

**FIGURE S3** Cell stemness is one of the determinants of the drug resistance to palbociclib in MCF‐7/R cell

**FIGURE S4** Saracatinib was identified as a candidate drug to reverse palbociclib resistance, but failed to achieve a greet effect

**FIGURE S5** PFKFB4 evoked metabolic reprogramming in ER^+^ breast cancer cell

**FIGURE S6** Interruption of the glycolysis pathway diminished cell stemness in ER^+^ breast cancer cell

**FIGURE S7** PFKFB4 influences the stemness and EMT of the breast cancer cell in vivo

**FIGURE S8** CCNE1 knockdown partially restored sensitivity to palbociclib in MCF‐7/R cells

**TABLE S1** Identification of 24 differential metabolites and their related information in cell supernatant

**TABLE S2** Identification of 18 differential metabolites and their related information between MCF‐7 and PFKFB4‐OE MCF‐7 cell groups

## Data Availability

All data are available via the corresponding author.
