# Supplementary figures and images for: PFKFB4 facilitates palbociclib resistance in oestrogen receptor‐positive breast cancer by enhancing stemness
Source: Cell Prolif. 2022 Sep 20;56(1):e13337. doi: 10.1111/cpr.13337 (PMC9816941; doi:10.1111/cpr.13337)

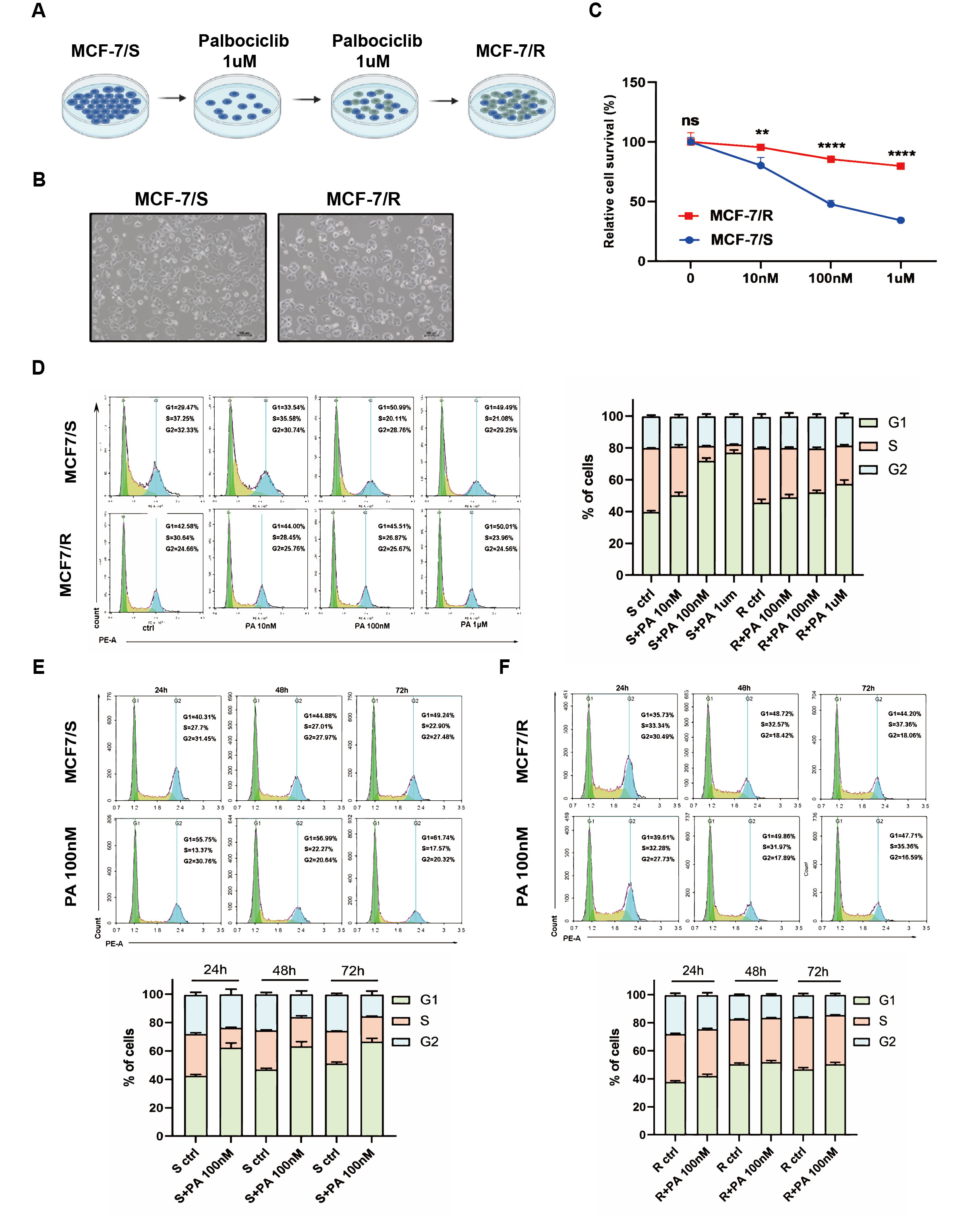

Supplement: Supplementary file 1 — FIGURE S1 Construction and evaluation of drug‐resistance ER+ breast cancer cell model [file CPR-56-e13337-s005.jpg]

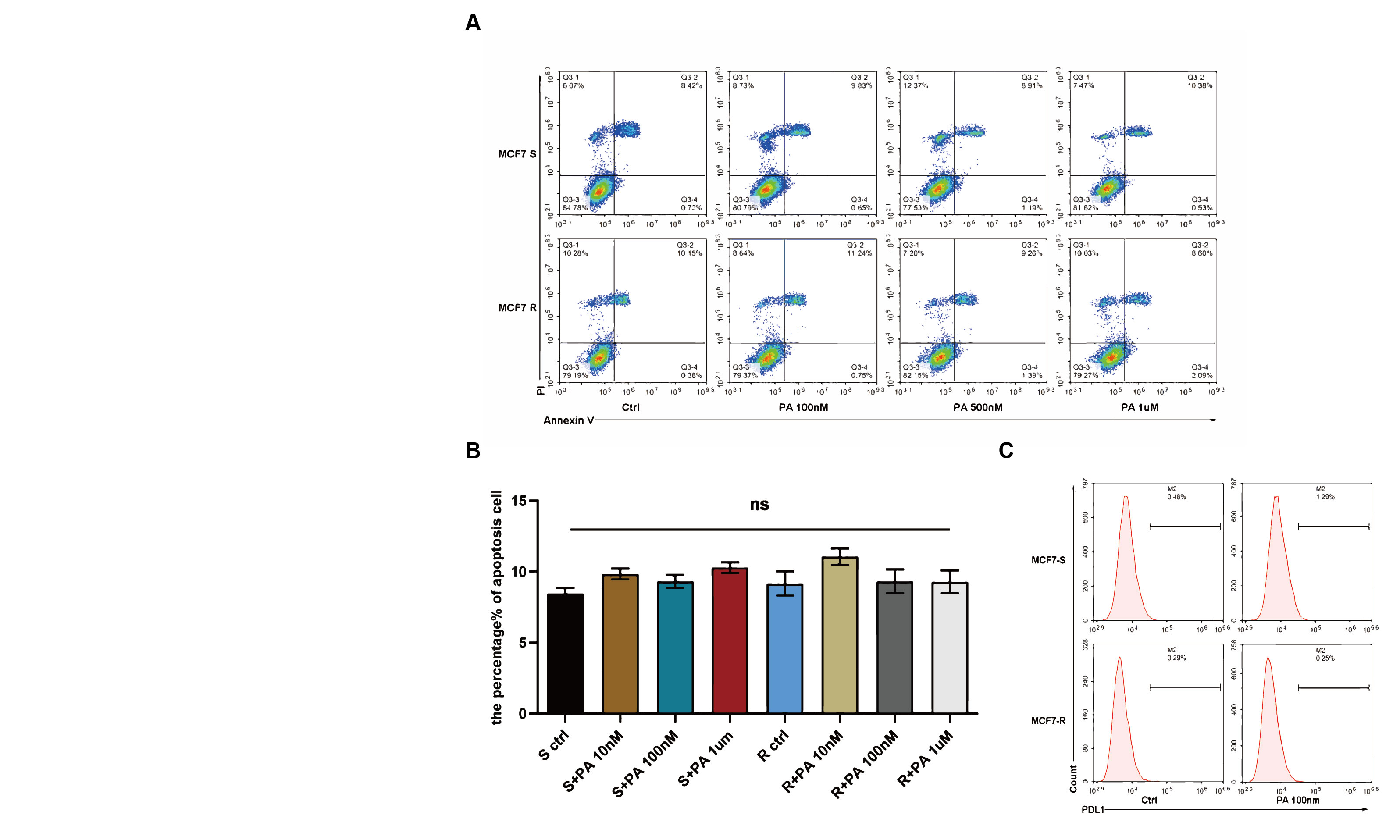

Supplement: Supplementary file 2 — FIGURE S2 Palbociclib caused no change in the expression of PD‐L1 and apoptosis levels in both MCF‐7/R and normal MCF‐7/S cells [file CPR-56-e13337-s004.jpg]

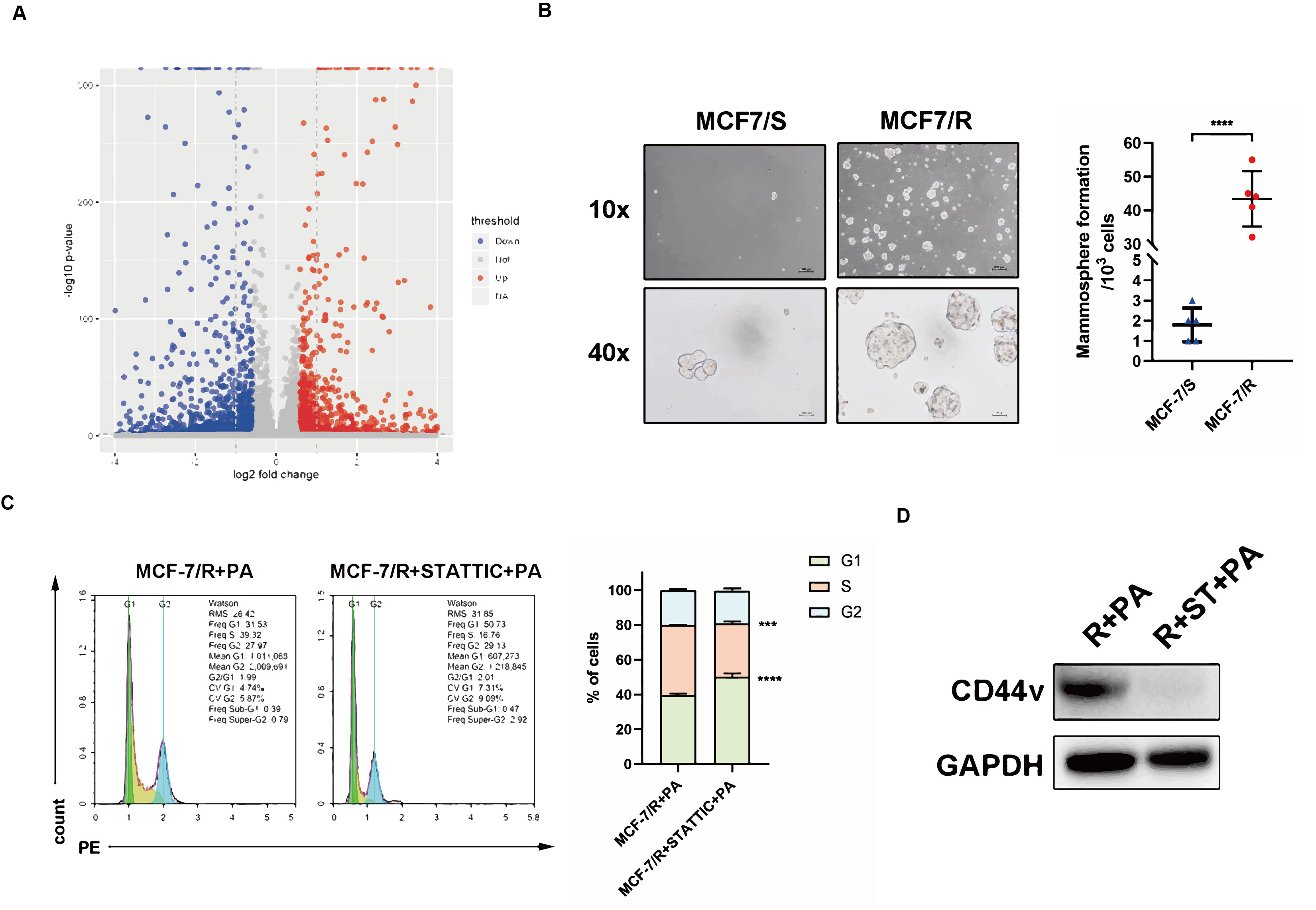

Supplement: Supplementary file 3 — FIGURE S3 Cell stemness is one of the determinants of the drug resistance to palbociclib in MCF‐7/R cell [file CPR-56-e13337-s009.jpg]

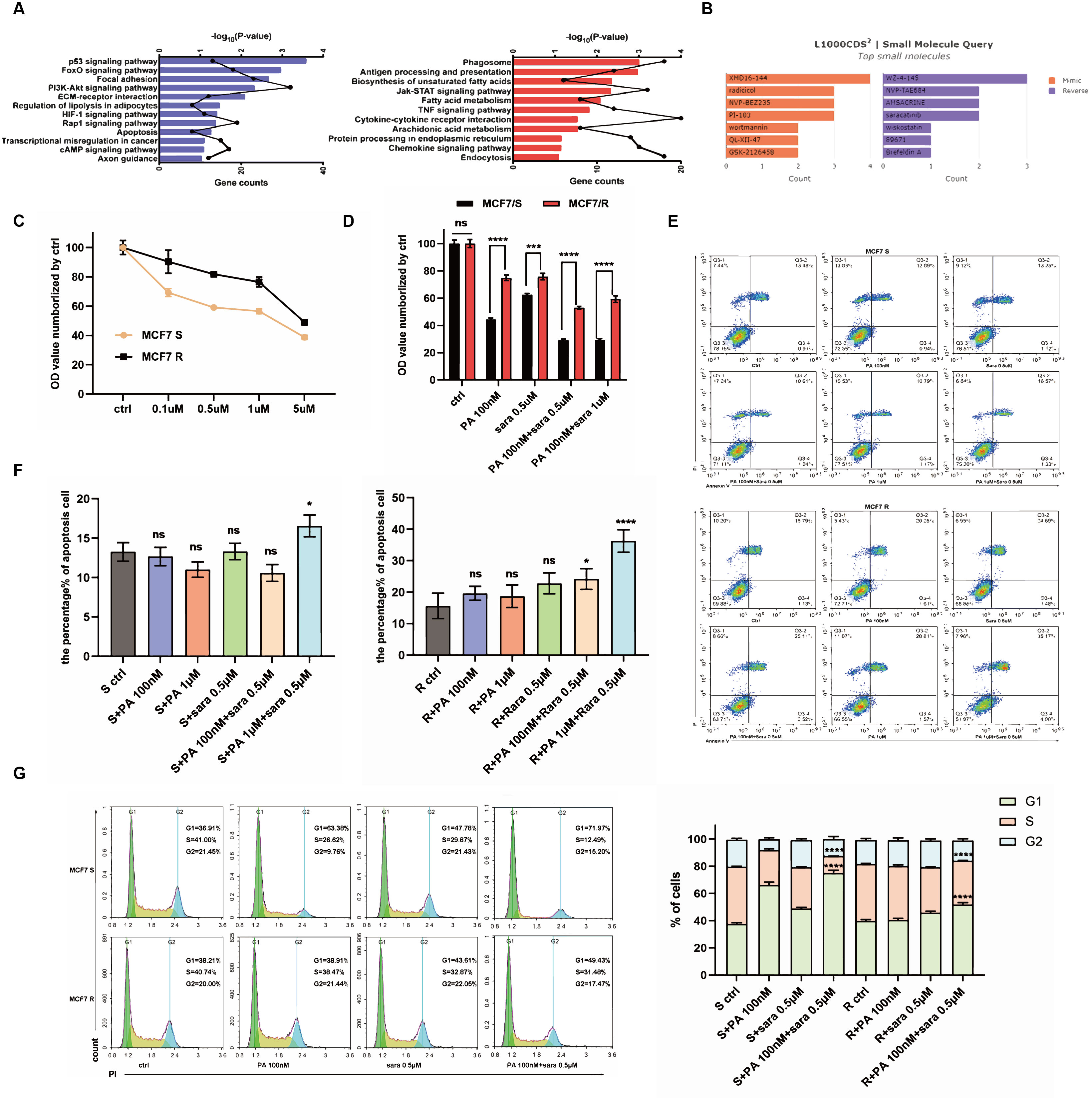

Supplement: Supplementary file 4 — FIGURE S4 Saracatinib was identified as a candidate drug to reverse palbociclib resistance, but failed to achieve a greet effect [file CPR-56-e13337-s002.jpg]

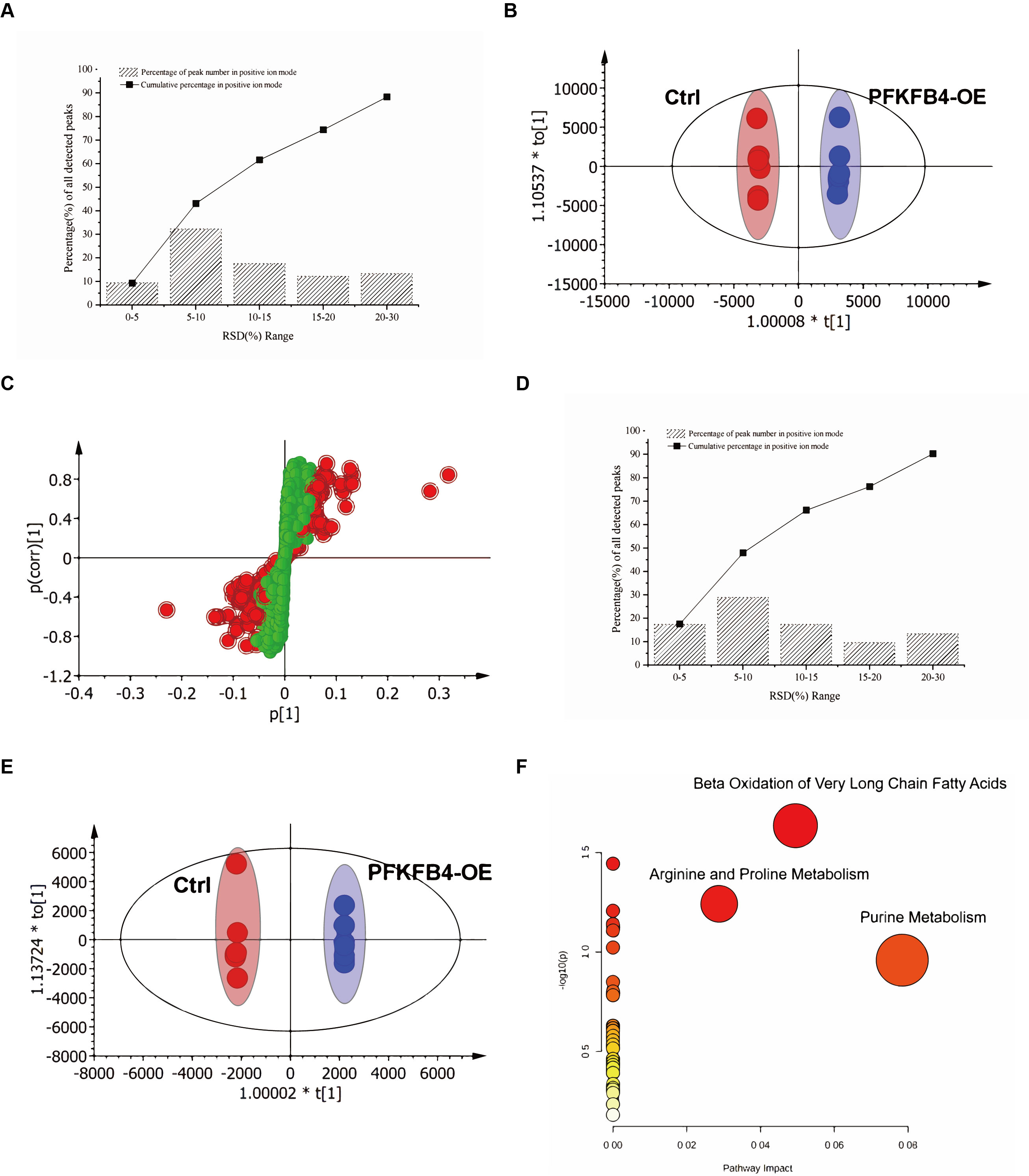

Supplement: Supplementary file 5 — FIGURE S5 PFKFB4 evoked metabolic reprogramming in ER+ breast cancer cell [file CPR-56-e13337-s006.jpg]

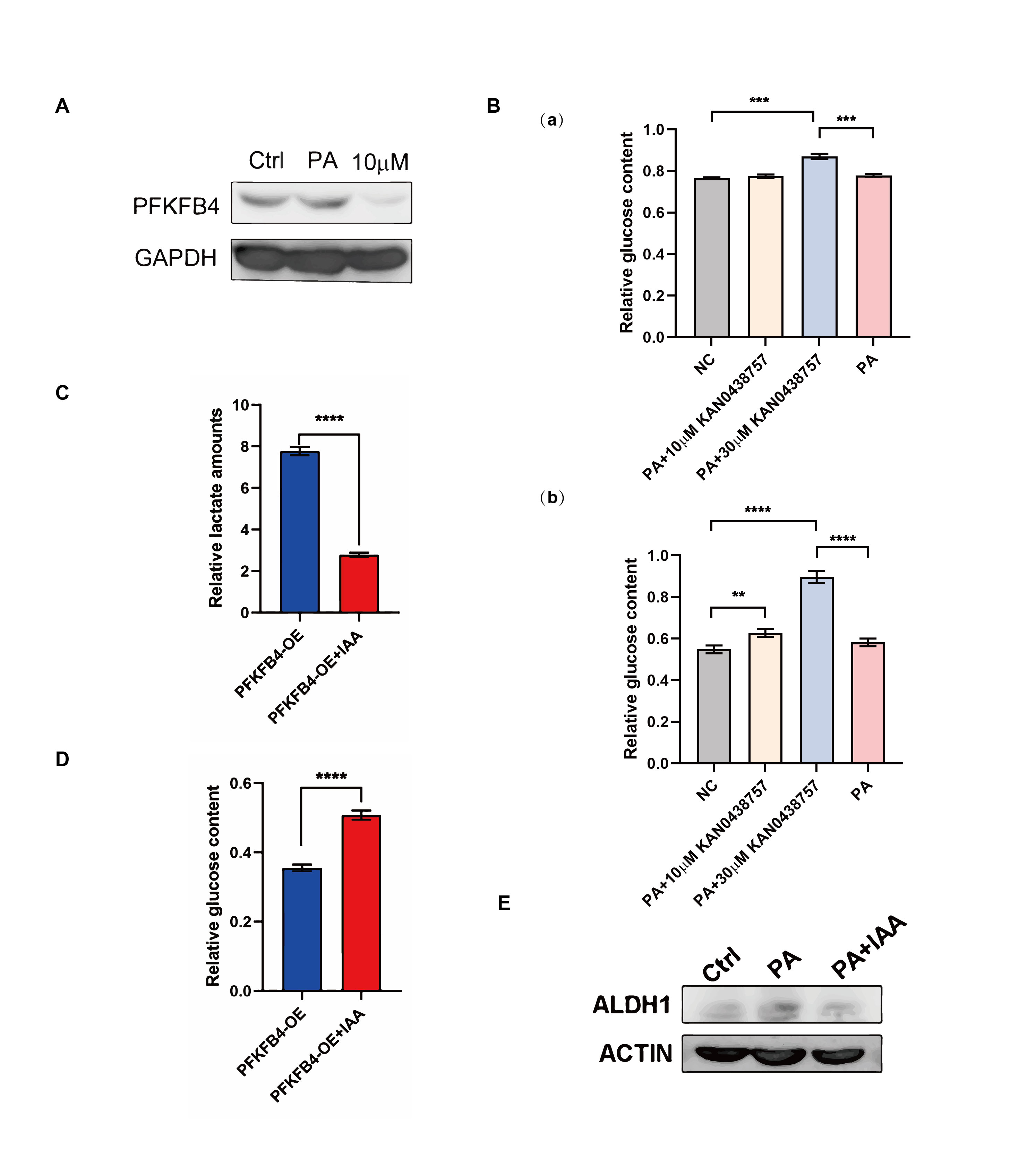

Supplement: Supplementary file 6 — FIGURE S6 Interruption of the glycolysis pathway diminished cell stemness in ER+ breast cancer cell [file CPR-56-e13337-s008.jpg]

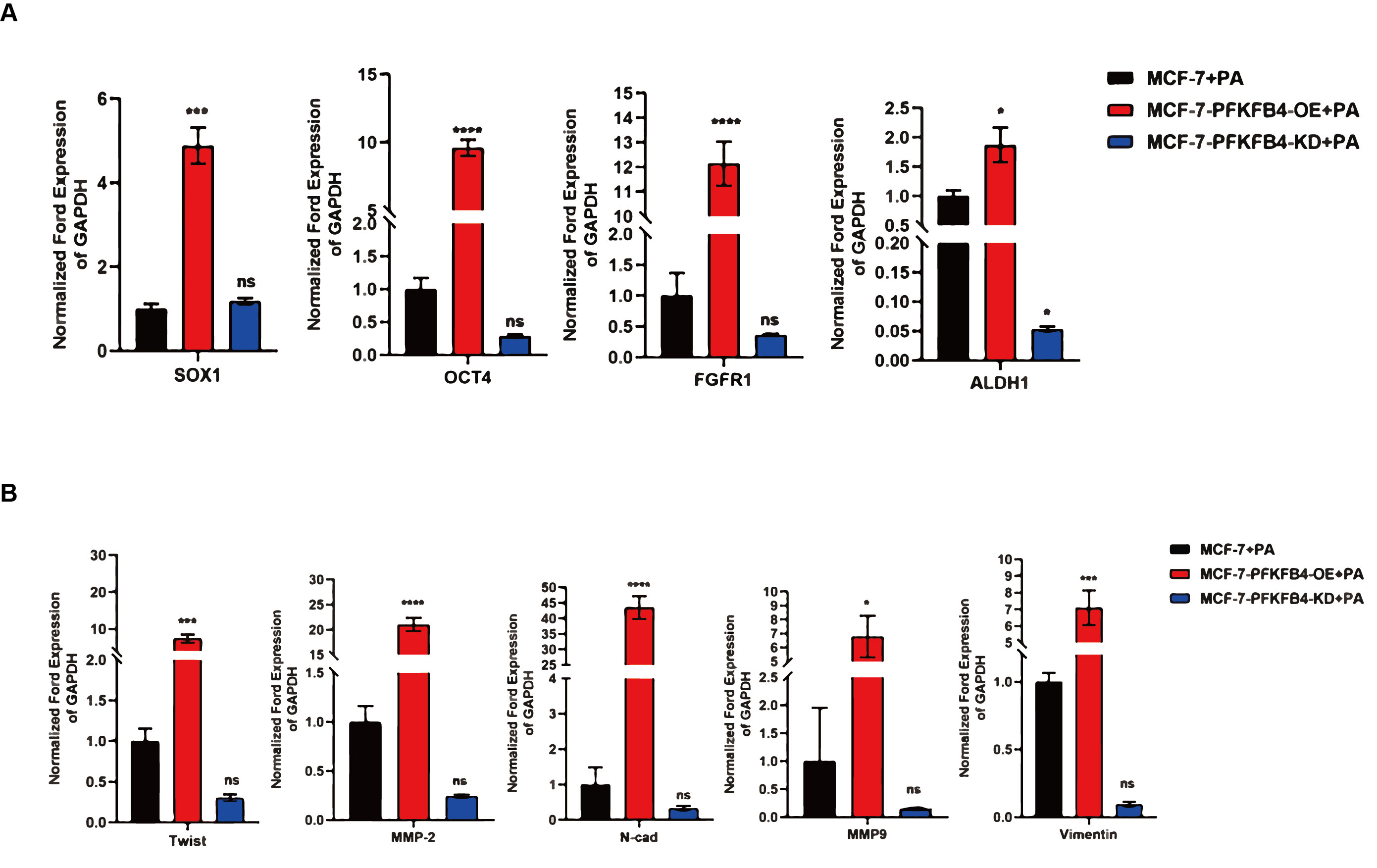

Supplement: Supplementary file 7 — FIGURE S7 PFKFB4 influences the stemness and EMT of the breast cancer cell in vivo [file CPR-56-e13337-s001.jpg]

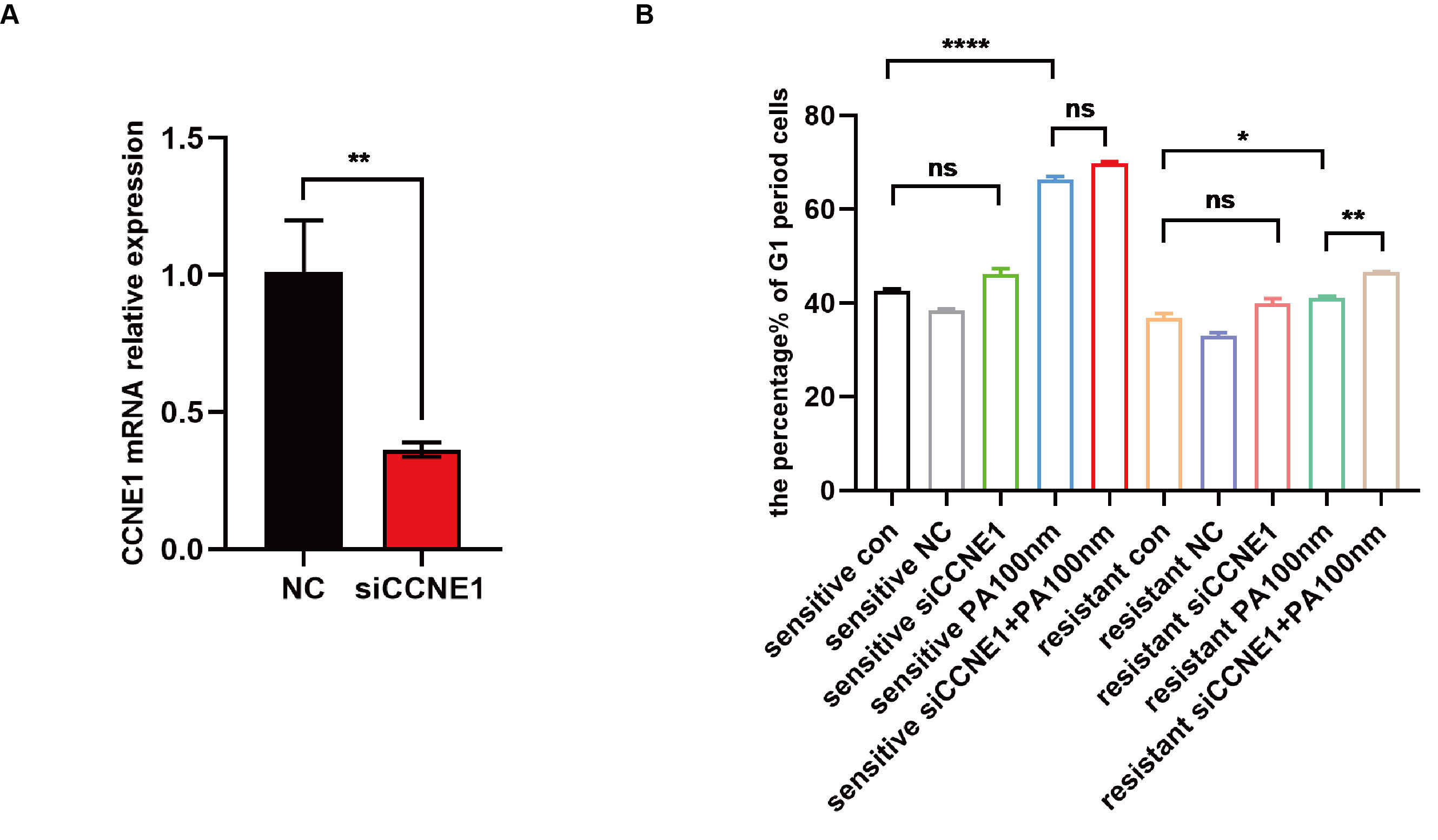

Supplement: Supplementary file 8 — FIGURE S8 CCNE1 knockdown partially restored sensitivity to palbociclib in MCF‐7/R cells [file CPR-56-e13337-s010.jpg]
