# Supplementary material for: PFKFB4 facilitates palbociclib resistance in oestrogen receptor‐positive breast cancer by enhancing stemness
Source: Cell Prolif. 2022 Sep 20;56(1):e13337. doi: 10.1111/cpr.13337 (PMC9816941; doi:10.1111/cpr.13337)
Supplement: Supplementary file 9 — TABLE S1 Identification of 24 differential metabolites and their related information in cell supernatant [file CPR-56-e13337-s007.docx]

**Identification of twenty-four differential metabolites and their related information in cell supernatant.**

| **ID** | **Retention**  **Time (min)** | **m/z**  **determined** | **m/z**  **calculated** | **Error**  **(ppm)** | **Ion form** | **Molecular**  **Formula** | **Compound**  **Name** |
| --- | --- | --- | --- | --- | --- | --- | --- |
| 1 | 0.824 | 90.948 | 90.9945 | -2.95 | M+H | C3H3ClO | cis-3-Chloroallyl aldehyde |
| 2 | 0.958 | 203.0524 | 203.0526 | 0.93 | M+Na | C6H12O6 | alpha-D-Glucose |
| 3 | 2.091 | 242.099 | 242.0999 | 1.23 | M+H | C9H17NO5 | Pantothenic acid |
| 4 | 1.129 | 130.0484 | 130.0475 | -7.06 | M+Na | C3H9NO3 | Ammonium lactate |
| 5 | 1.131 | 148.0964 | 148.0968 | 2.93 | M+H | C6H13NO3 | Fagomine |
| 6 | 1.147 | 198.0852 | 19832849 | -1.52 | M+Na | C6H13N3O3 | Citrulline |
| 7 | 1.150 | 182.0809 | 182.0812 | 1.31 | M+H | C9H11NO3 | L-Tyrosine |
| 8 | 1.267 | 276.1444 | 276.1442 | -0.68 | M+H | C12H21NO6 | Glutarylcarnitine |
| 9 | 1.287 | 132.1019 | 132.1019 | -0.19 | M+H | C6H13NO2 | L-Leucine |
| 10 | 1.488 | 160.0755 | 160.0757 | 1.09 | M+H | C10H9NO | Indoleacetaldehyde |
| 11 | 1.790 | 166.0861 | 166.0863 | 1.05 | M+H | C9H11NO2 | L-Phenylalanine |
| 12 | 0.958 | 203.0519 | 203.0526 | 3.49 | M+Na | C6H12O6 | Beta-D-Glucose |
| 13 | 2.453 | 232.1544 | 232.1543 | -0.46 | M+H | C11H21NO4 | Butyrylcarnitine |
| 14 | 7.591 | 335.1061 | 335.106 | -0.20 | M+H | C16H18N2O4S | Penicillin G |
| 15 | 8.238 | 355.1167 | 355.1176 | 2.71 | M+H | C20H18O6 | Glyceofuran |
| 16 | 8.502 | 450.1689 | 450.1687 | -0.30 | M+Na | C24H26FNO5 | 6-Hydroxyfluvastatin |
| 17 | 9.254 | 367.1874 | 367.188 | 1.65 | M+Na | C21H28O4 | 11-Dehydrocorticosterone |
| 18 | 9.821 | 244.1905 | 244.1907 | 0.90 | M+H | C13H25NO3 | N-Undecanoylglycine |
| 19 | 10.737 | 318.2999 | 318.3003 | 1.14 | M+H | C18H39NO3 | Phytosphingosine |
| 20 | 12.320 | 372.3113 | 372.3108 | -1.14 | M+H | C21H41NO4 | Tetradecanoylcarnitine |
| 21 | 13.504 | 426.3576 | 426.3578 | 0.43 | M+H | C25H47NO4 | Vaccenyl carnitine |
| 22 | 14.170 | 279.1856 | 279.1856 | 0.07 | M+H | C19H22N2 | Triprolidine |
| 23 | 14.186 | 149.0260 | 149.0267 | 4.62 | M+H | C5H8O3S | 2-Oxo-4-methylthiobutanoic acid |
| 24 | 14.187 | 280.1674 | 280.1672 | -0.63 | M+Na | C17H23NO | Levorphanol |
