# Supplementary material for: PFKFB4 facilitates palbociclib resistance in oestrogen receptor‐positive breast cancer by enhancing stemness
Source: Cell Prolif. 2022 Sep 20;56(1):e13337. doi: 10.1111/cpr.13337 (PMC9816941; doi:10.1111/cpr.13337)
Supplement: Supplementary file 10 — TABLE S2 Identification of 18 differential metabolites and their related information between MCF‐7 and PFKFB4‐OE MCF‐7 cell groups [file CPR-56-e13337-s003.docx]

**Identification of eighteen differential metabolites and their related information between MCF-7 and PFKFB4-OE MCF-7 cell groups.**

| **ID** | **Retention**  **Time (min)** | **m/z**  **determined** | **m/z**  **calculated** | **Error**  **(ppm)** | **Ion form** | **Molecular**  **Formula** | **Compound**  **Name** |
| --- | --- | --- | --- | --- | --- | --- | --- |
| 1 | 0.871 | 140.0679 | 140.0682 | 2.41 | M+Na | C5H11NO2 | L-Valine |
| 2 | 0.905 | 162.1125 | 162.1125 | -0.41 | M+H | C7H15NO3 | L-Carnitine |
| 3 | 0.956 | 132.0766 | 132.0768 | 1.15 | M+H | C4H9N3O2 | Creatine |
| 4 | 0.968 | 348.0698 | 348.0704 | 1.6 | M+H | C10H14N5O7P | Adenosine 2'-phosphate |
| 5 | 0.973 | 116.0710 | 116.0706 | -3.2 | M+H | C5H9NO2 | D-Proline |
| 6 | 0.989 | 201.1230 | 204.123 | 0.21 | M+H | C9H17NO4 | L-Acetylcarnitine |
| 7 | 0.990 | 258.1182 | 258.1197 | 5.77 | M+H | C9H15N5O4 | 4a-Hydroxytetrahydrobiopterin |
| 8 | 0.991 | 102.1278 | 102.1277 | -0.39 | M+H | C6H15N | Triethylamine |
| 9 | 1.040 | 268.1044 | 268.104 | -1.4 | M+H | C10H13N5O4 | Adenosine |
| 10 | 1.041 | 137.0463 | 137.0458 | -3.62 | M+H | C5H4N4O | Hypoxanthine |
| 11 | 1.110 | 132.1022 | 132.1019 | -2.02 | M+H | C6H13NO2 | L-Leucine |
| 12 | 1.749 | 232.1541 | 232.1543 | 1.02 | M+H | C11H21NO4 | Butyrylcarnitine |
| 13 | 7.582 | 372.3107 | 372.3108 | 0.27 | M+H | C21H41NO4 | Tetradecanoylcarnitine |
| 14 | 8.059 | 400.3416 | 400.3421 | 1.34 | M+H | C23H45NO4 | L-Palmitoylcarnitine |
| 15 | 8.181 | 426.3572 | 426.3578 | 1.4 | M+H | C25H47NO4 | Oleoylcarnitine |
| 16 | 8.423 | 255.1492 | 255.1492 | -0.19 | M+H | C16H18N2O | (+)-Setoclavine |
| 17 | 8.957 | 279.1588 | 279.1591 | 0.86 | M+H | C16H22O4 | Diisobutyl phthalate |
| 18 | 15.175 | 122.0965 | 122.0694 | -0.34 | M+H | C8H11N | 1-Phenylethylamine |
